# Supplementary material for: Synthesis of Homodrimane Sesquiterpenoids Bearing 1,3-Benzothiazole Unit and Their Antimicrobial Activity Evaluation
Source: Molecules. 2022 Aug 10;27(16):5082. doi: 10.3390/molecules27165082 (PMC9414590; doi:10.3390/molecules27165082)

Synthesis of Homodrimane Sesquiterpenoids Bearing 1,3-Benzothiazole Unit and Their Antimicrobial Activity Evaluation

Lidia Lungu [1], Caleria Cucicova [1], Svetlana Blaja [1], Alexandru Ciocarlan [1], Ion Dragalin [1], Alic Barba [1], Nicoleta Vornicu [2], Elisabeta-Irina Geana [3], Ionel I. Mangalagiu [4] and Aculina Aricu [1,*]

1 Chemistry of Natural and Biologically Active Compounds Laboratory, Institute of Chemistry, 3 Academiei Str., MD-2028 Chisinau, Moldova; lidilungu@yahoo.com (L.L.); cucicovac@yahoo.com (C.C.); svetlana-blaja@mail.ru (S.B.); algciocarlan@yahoo.com (A.C.); iondragalin@yahoo.com (I.D.);

2 Metropolitan Center of Research T.A.B.O.R., 9 Closca Str., RO-700066 Iasi, Romania; cmctaboriasi@yahoo.com (N.V.)

3 Department of Research and Development, National Research and Development Institute for Cryogenics and Isotopic Technologies—ICSI Rm. Valcea, 4th Uzinei Str., PO Raureni Box 7, 240050 Rm. Valcea, Romania; irina.geana@icsi.ro (E.-I.G.)

4 Faculty of Chemistry, "*Alexandru Ioan Cuza*" University of Iasi, 11 Carol Bd., RO-700506 Iasi, Romania; ionelm@uaic.ro (I.I.M.)

* Correspondence: aculina.aricu@gmail.com (A.A.)

SUPPLEMENTARY INFORMATION

^{1}H NMR spectrum of compound 3

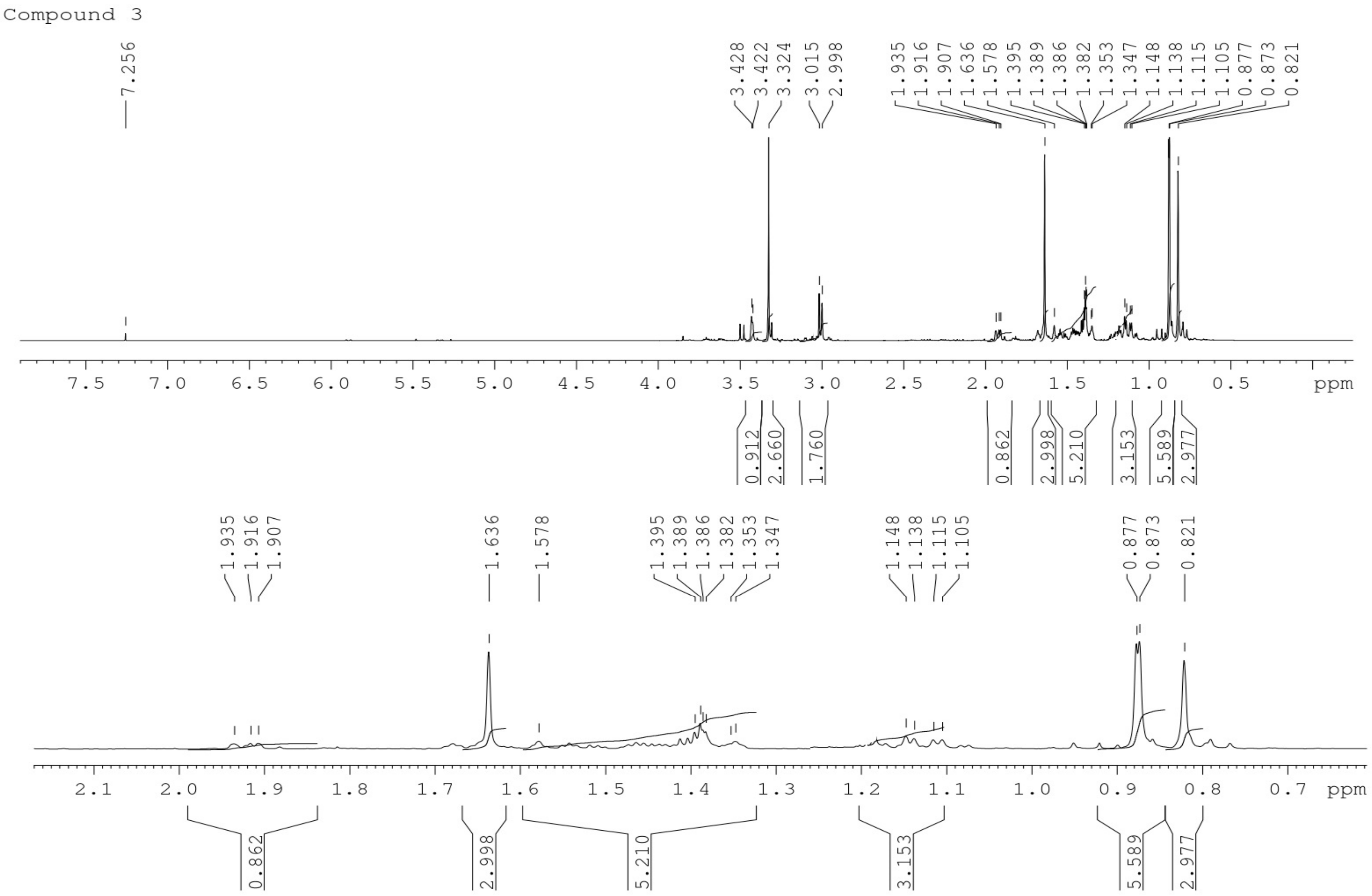

^{13}C NMR spectrum of compound 3

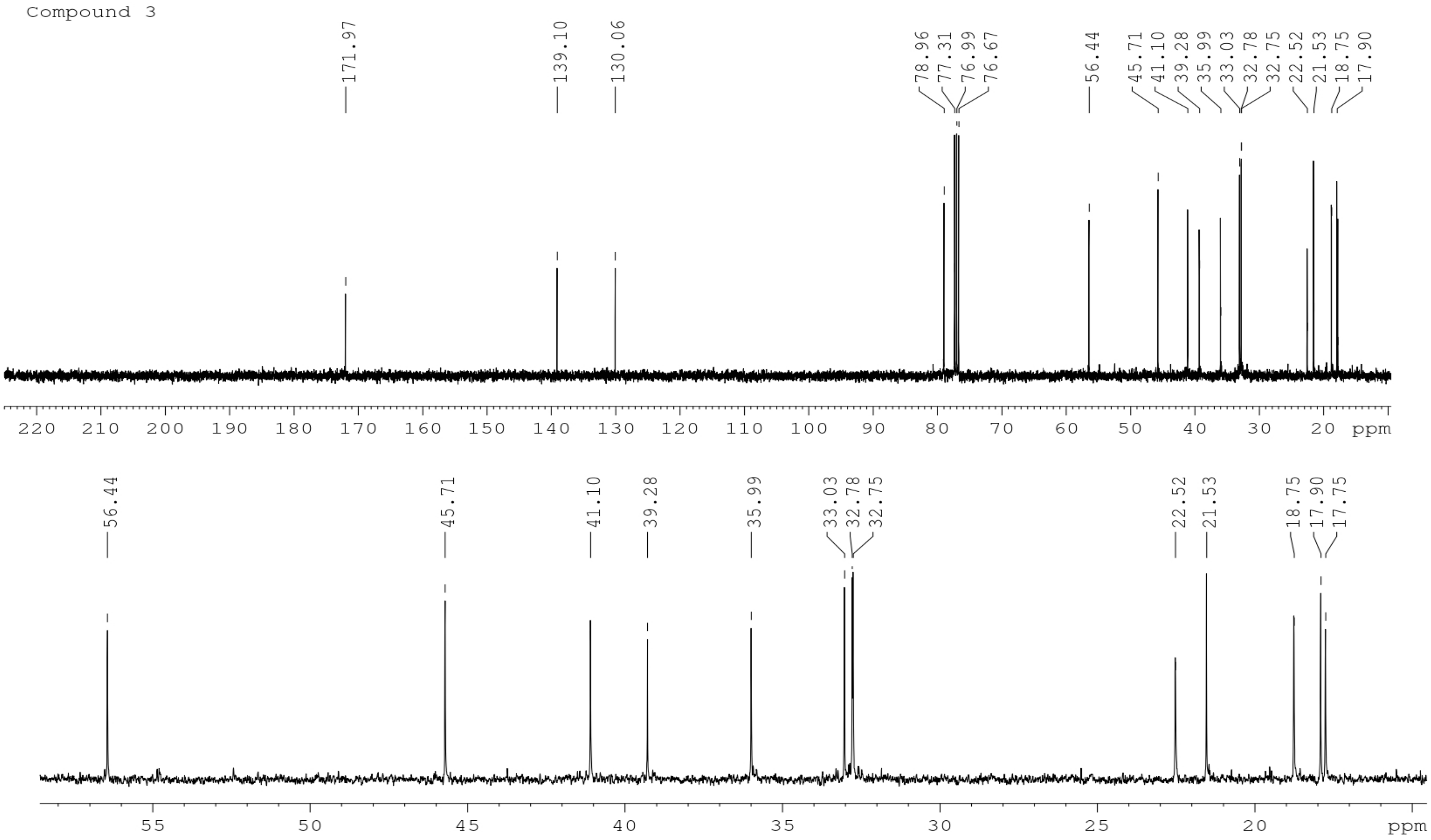

^{1}H NMR spectrum of compound 4

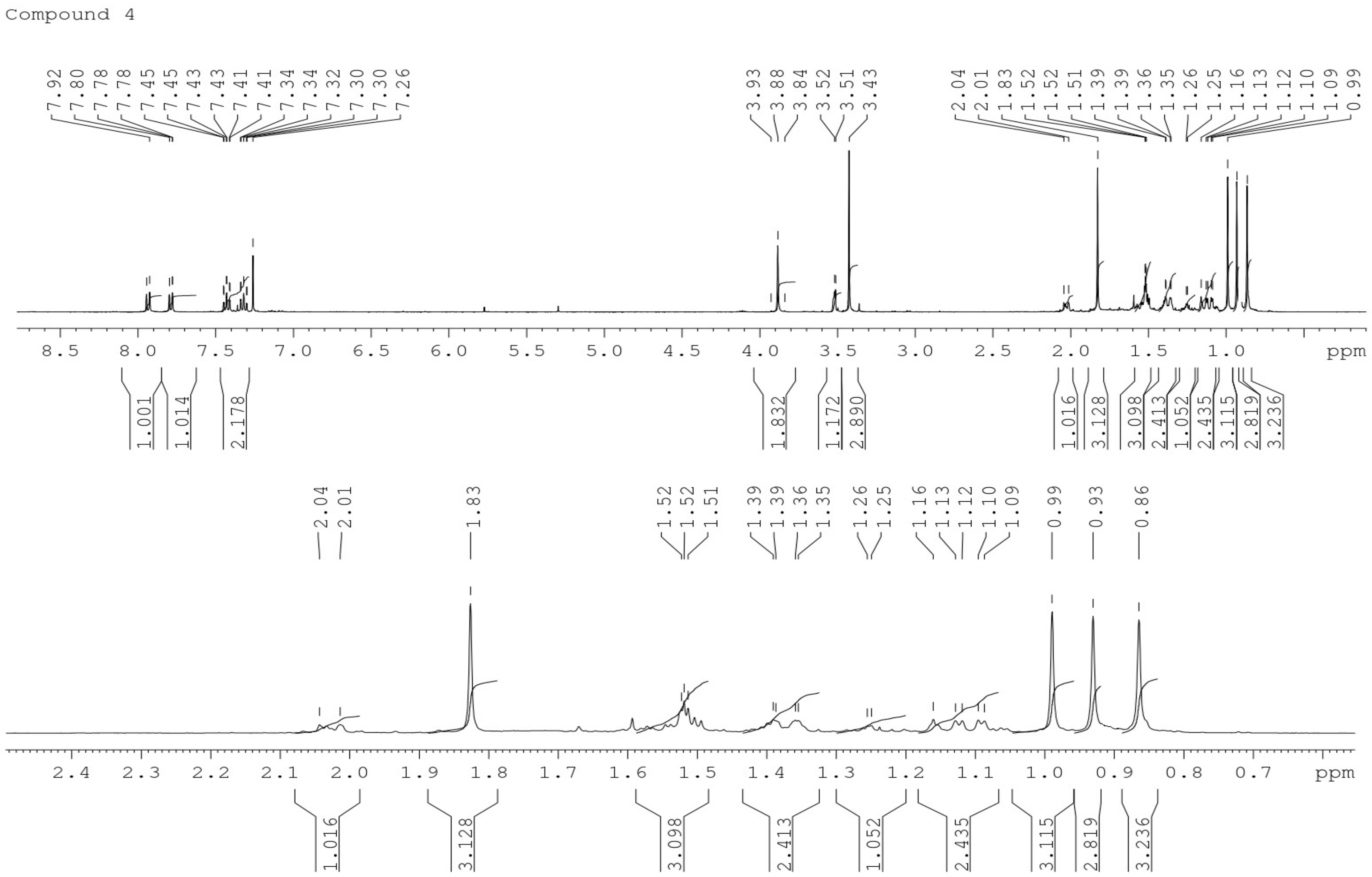

^{13}C NMR spectrum of compound 4

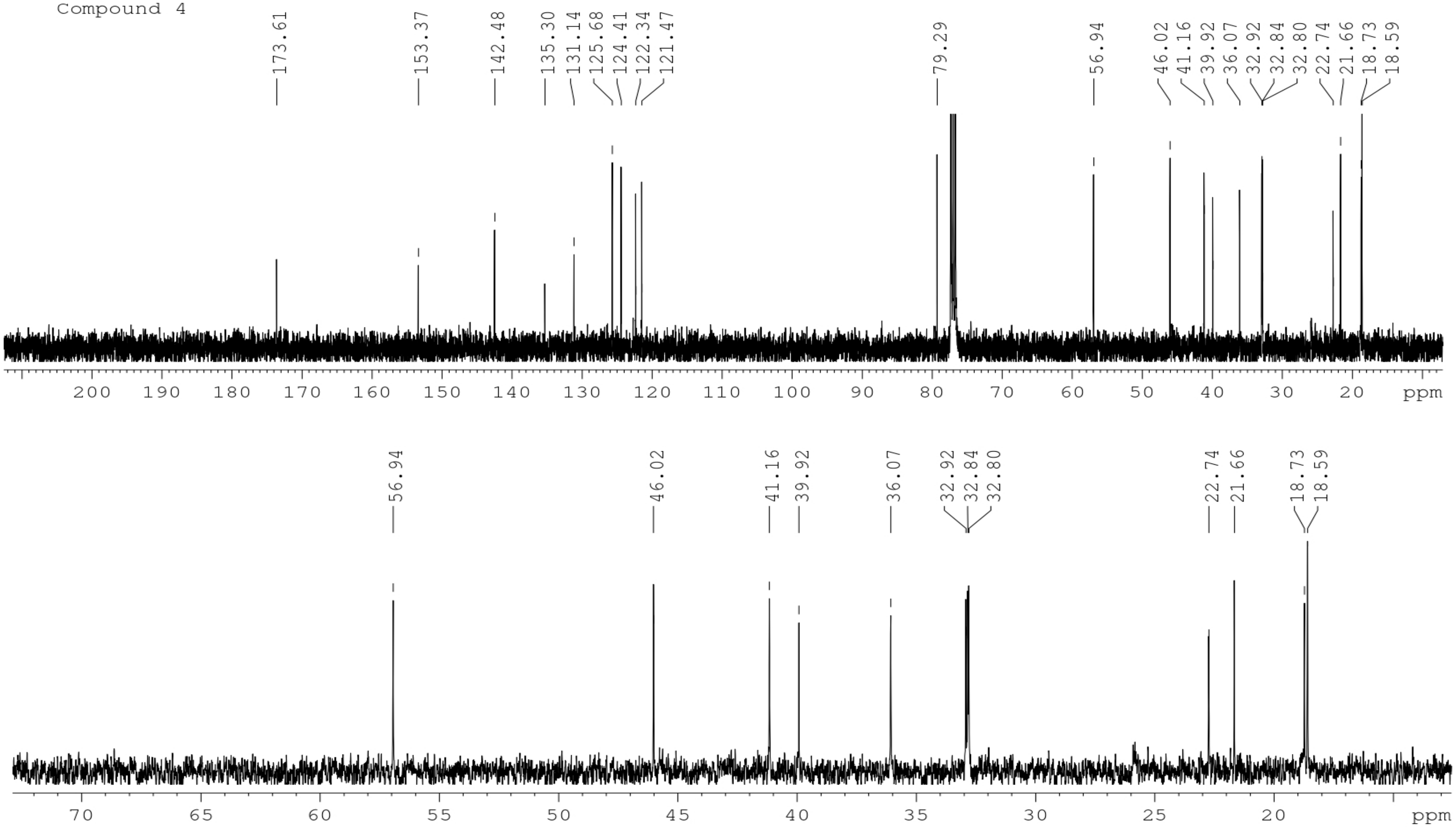

^{1}H NMR spectrum of compound 6

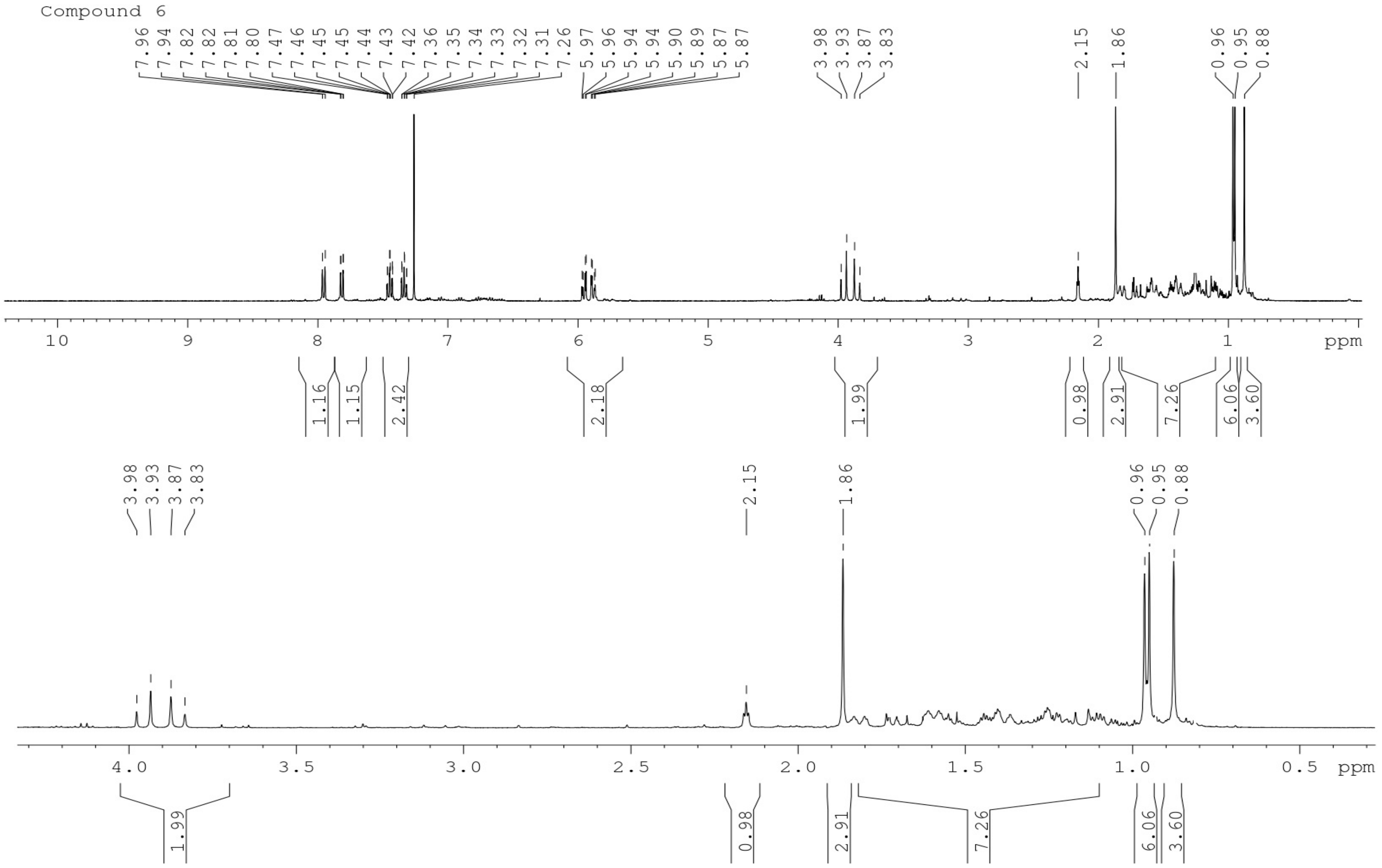

^{13}C NMR spectrum of compound 6

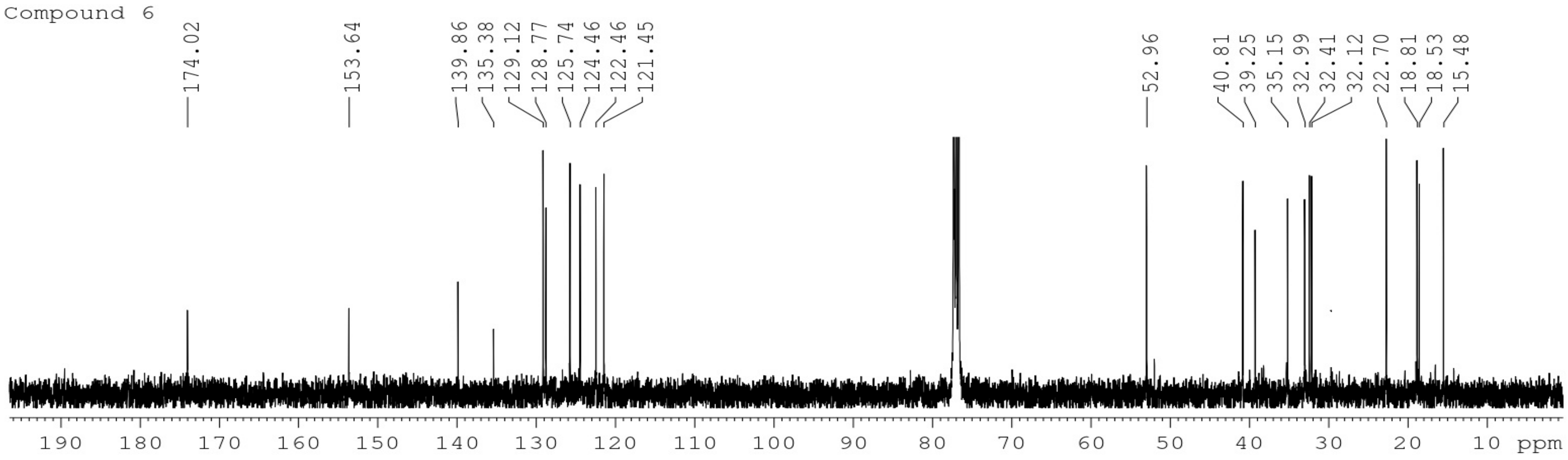

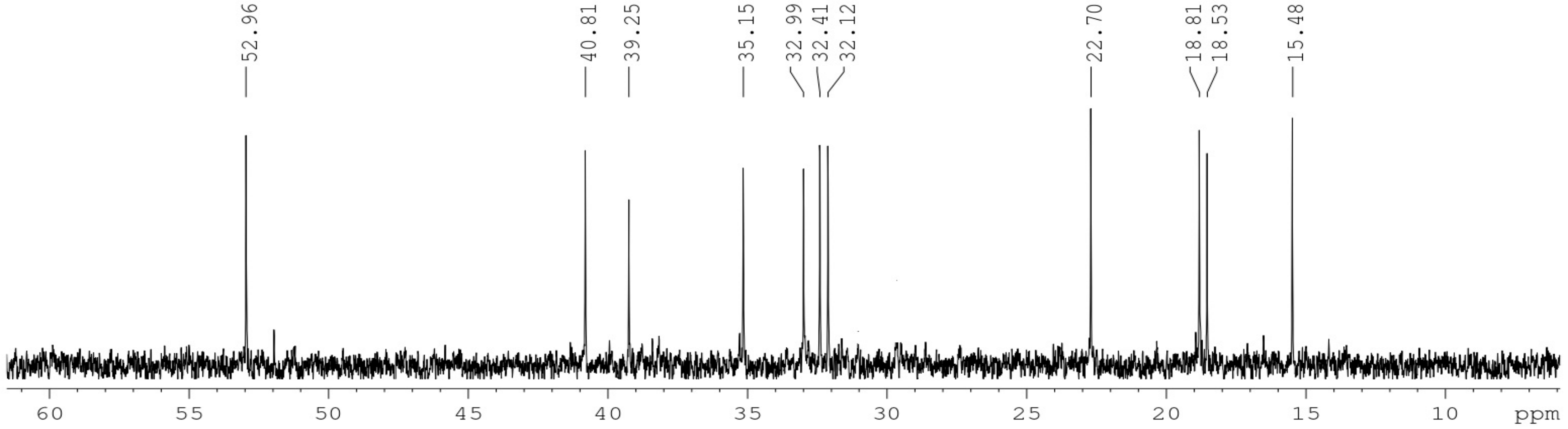

^{1}H NMR spectrum of compound 8

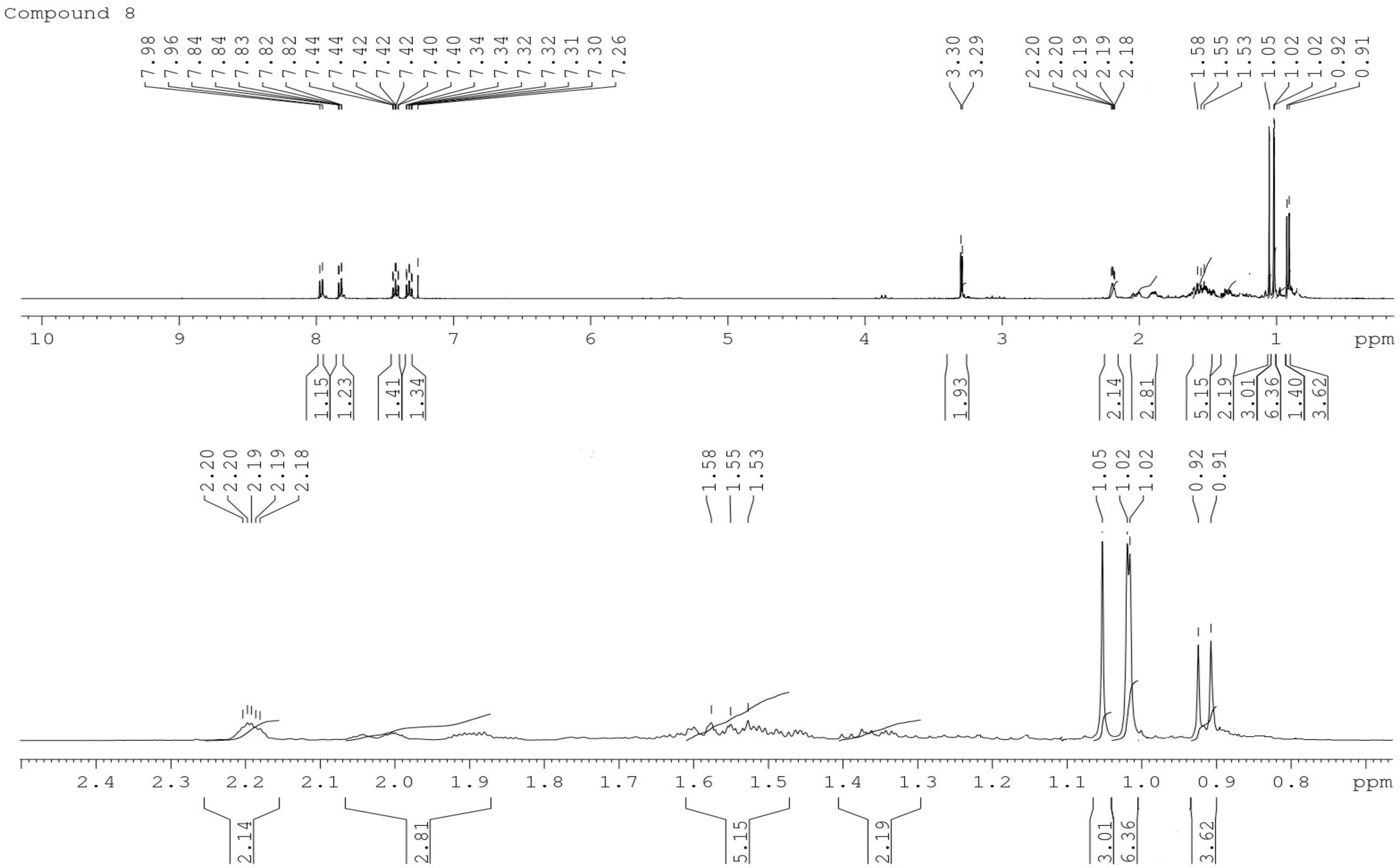

^{13}C NMR spectrum of compound 8

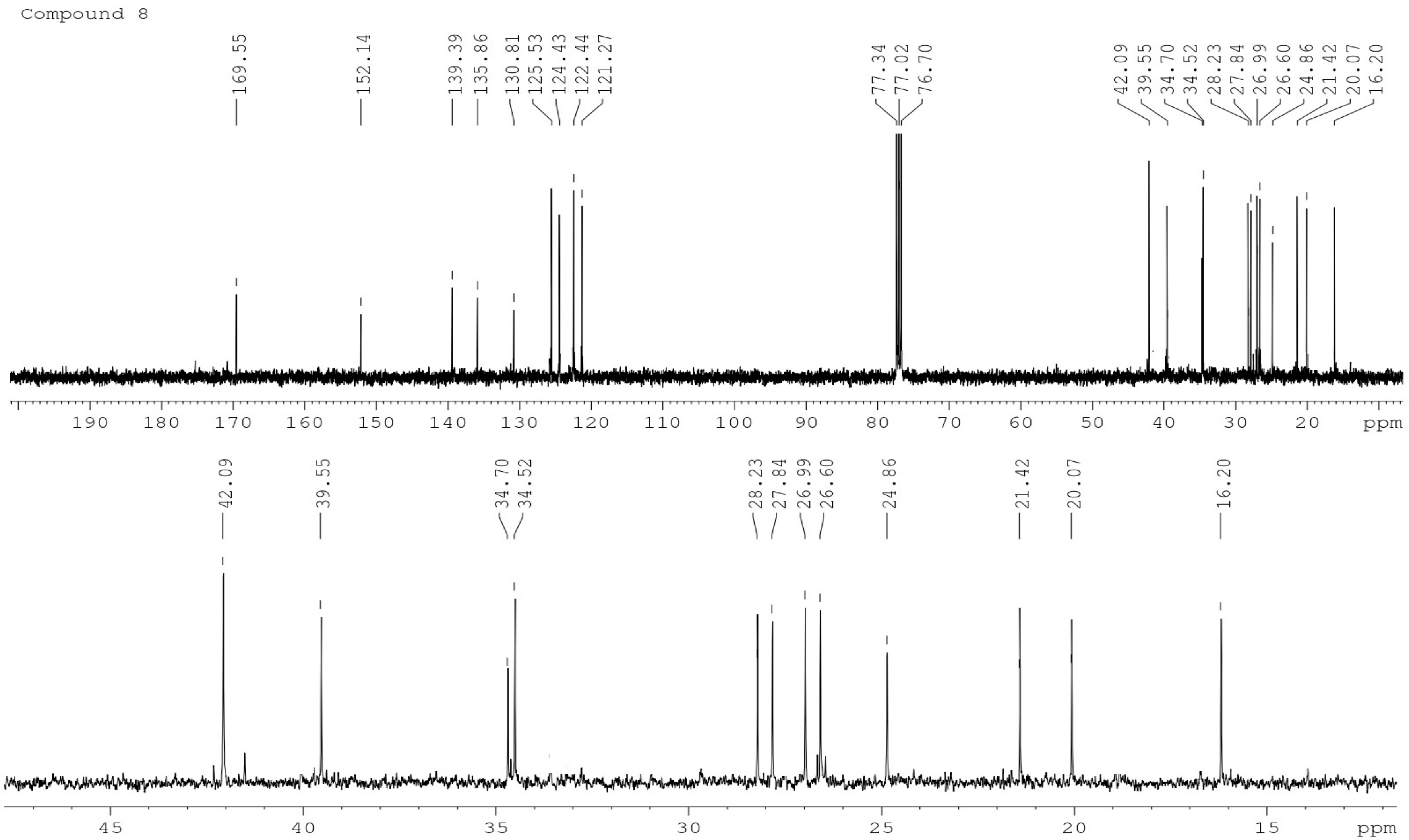

^{1}H NMR spectrum of compound 9

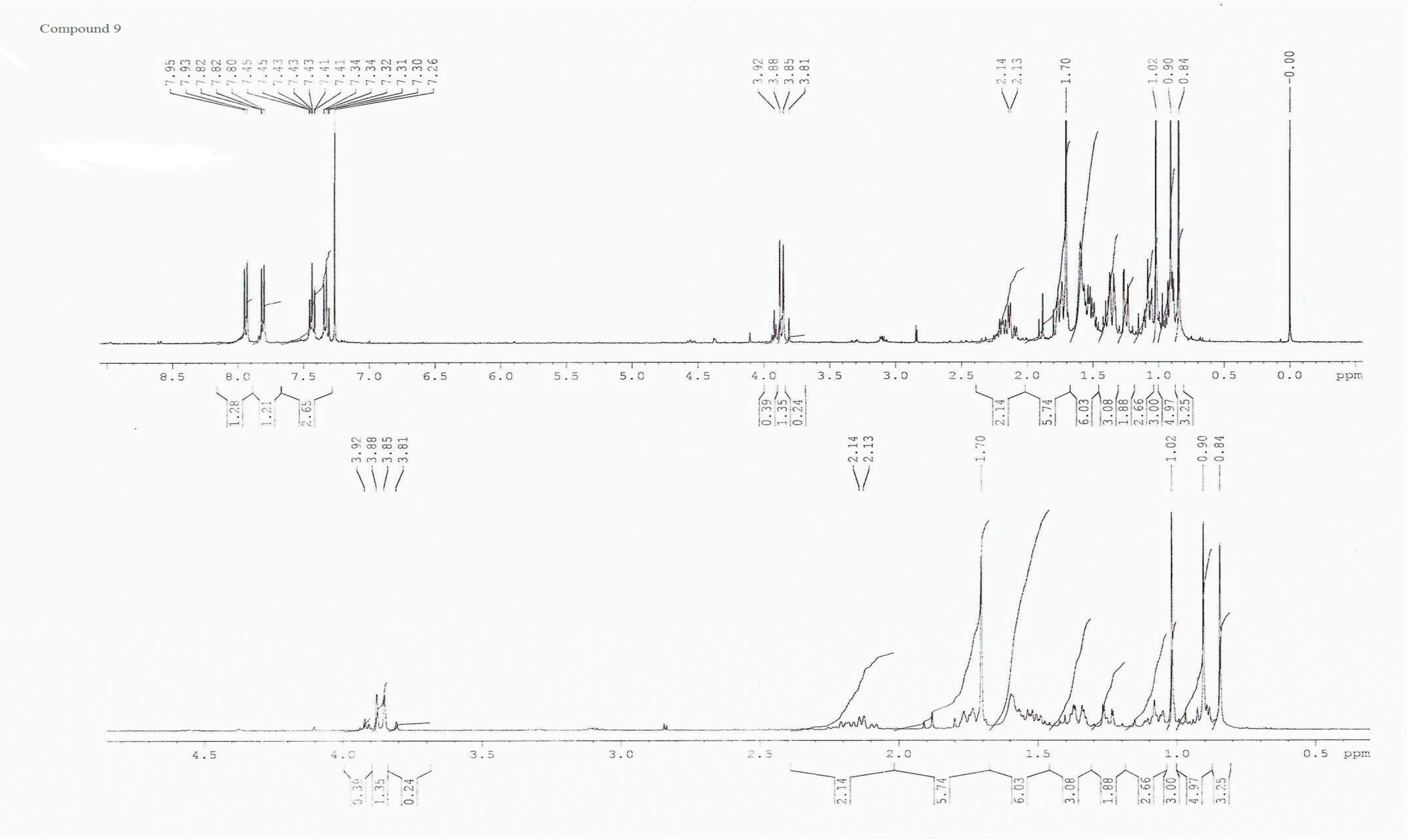

^{13}C NMR spectrum of compound 9

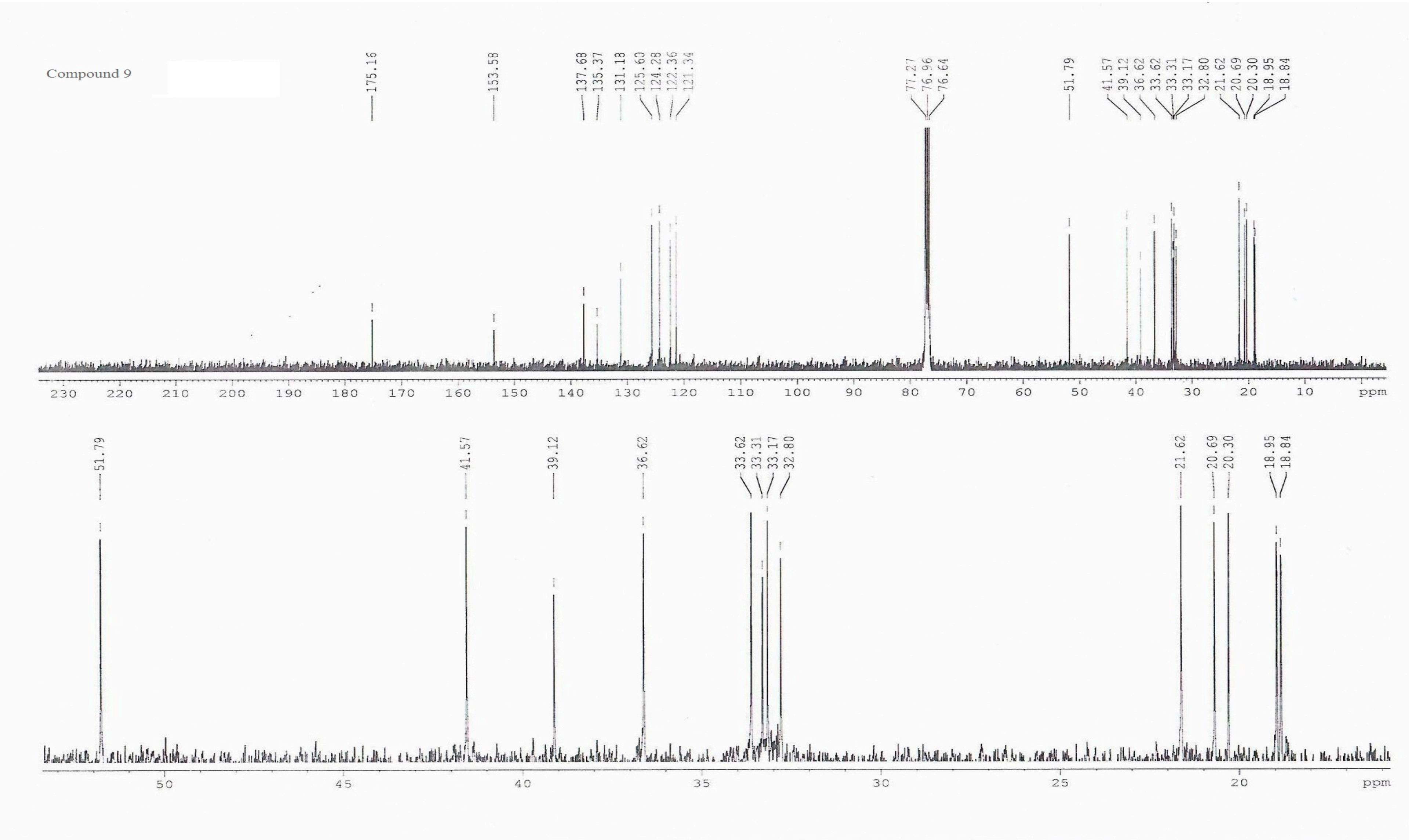

^{1}H NMR spectrum of compound 14

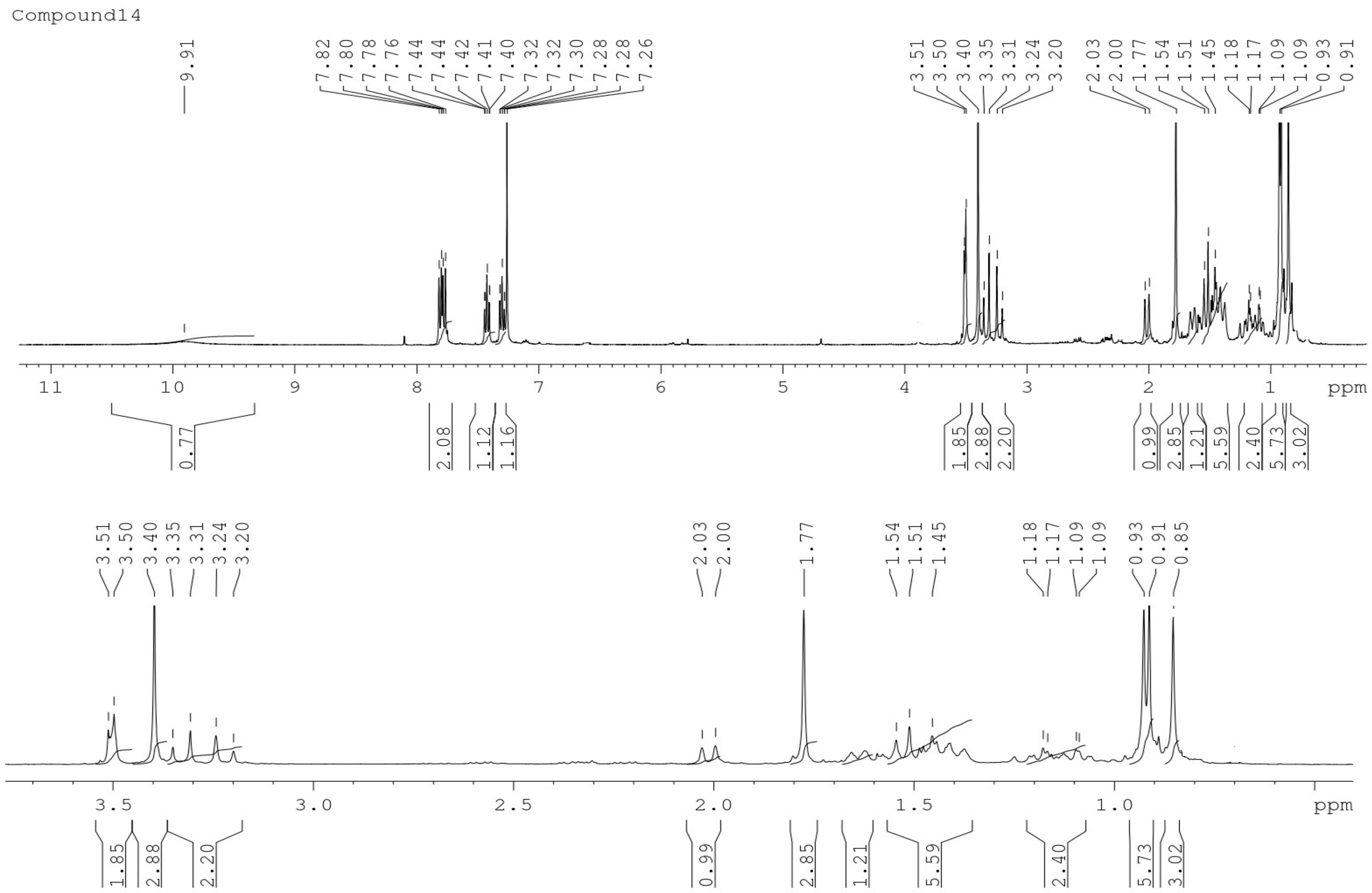

^{13}C NMR spectrum of compound 14

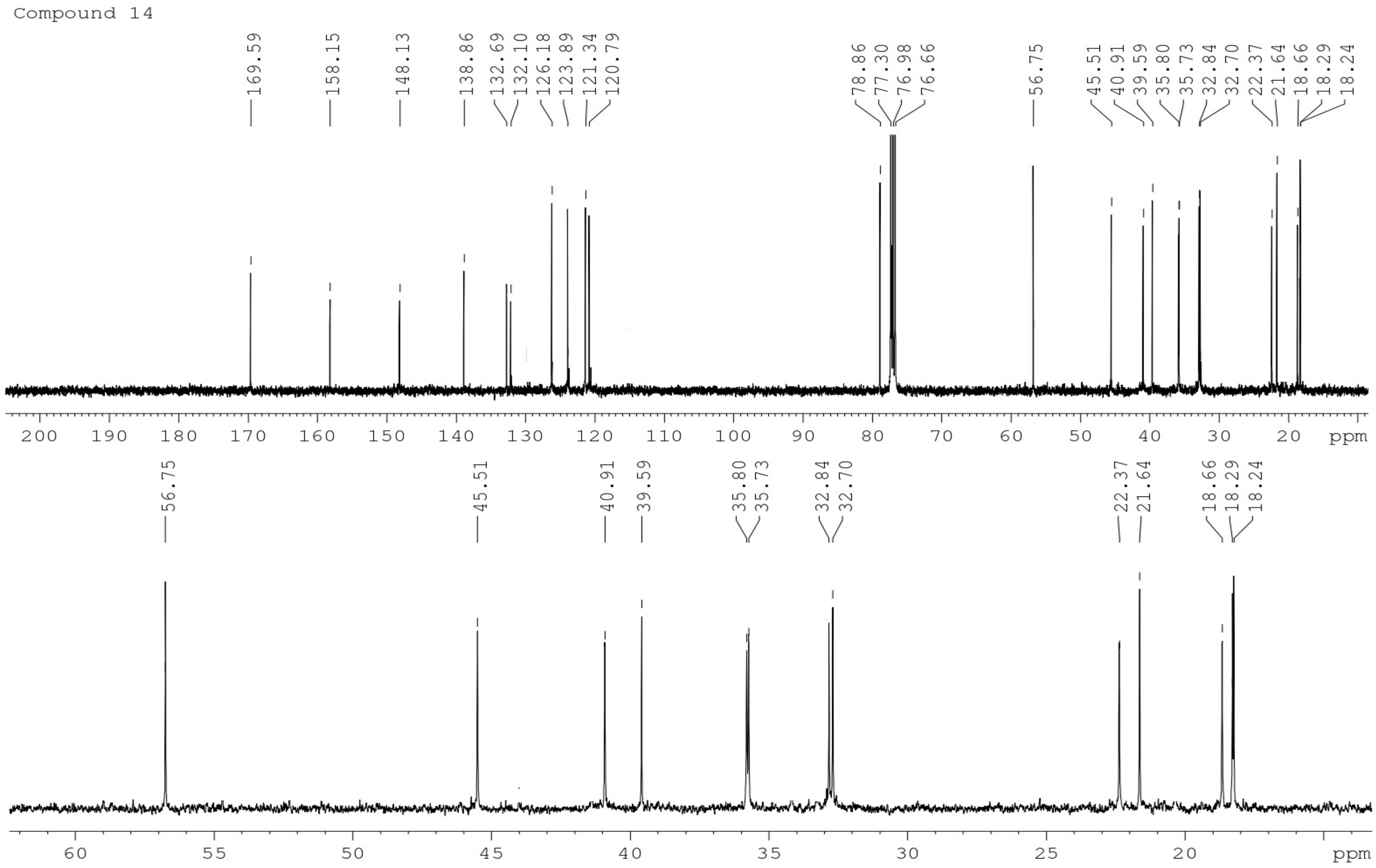

^{1}H NMR spectrum of compound 16

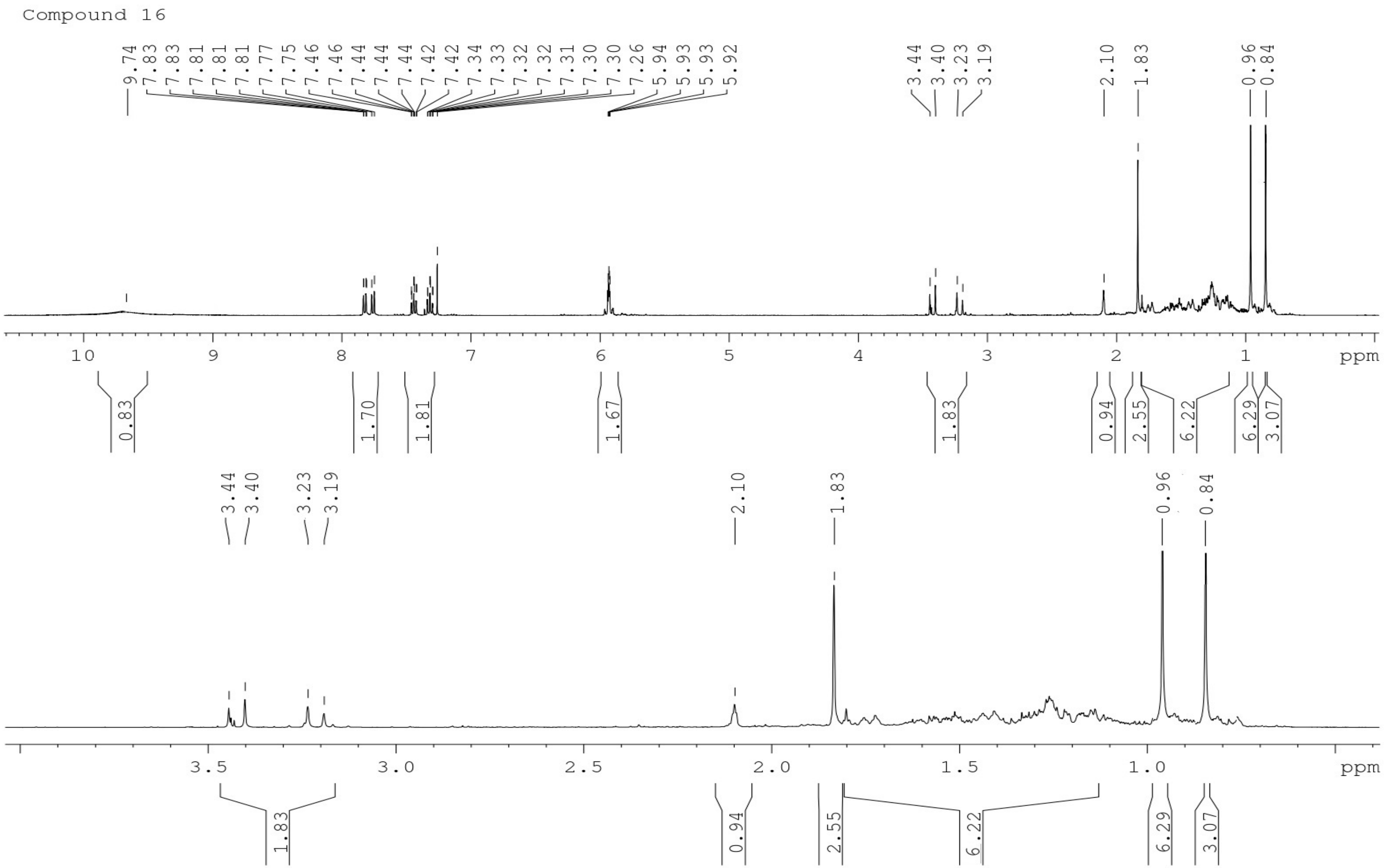

^{13}C NMR spectrum of compound 16

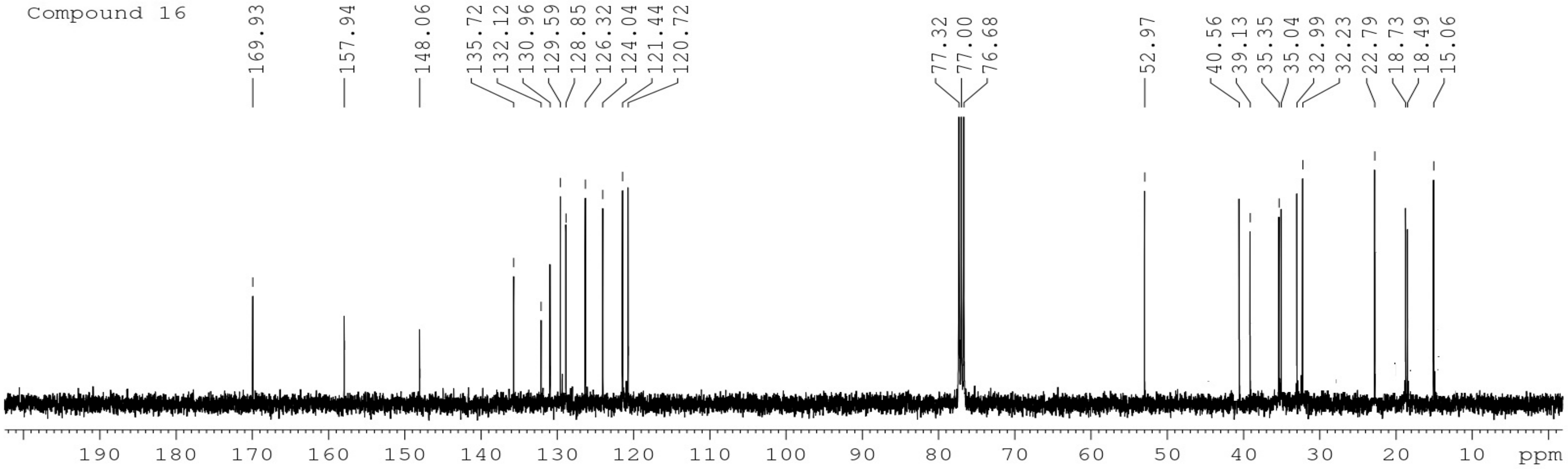

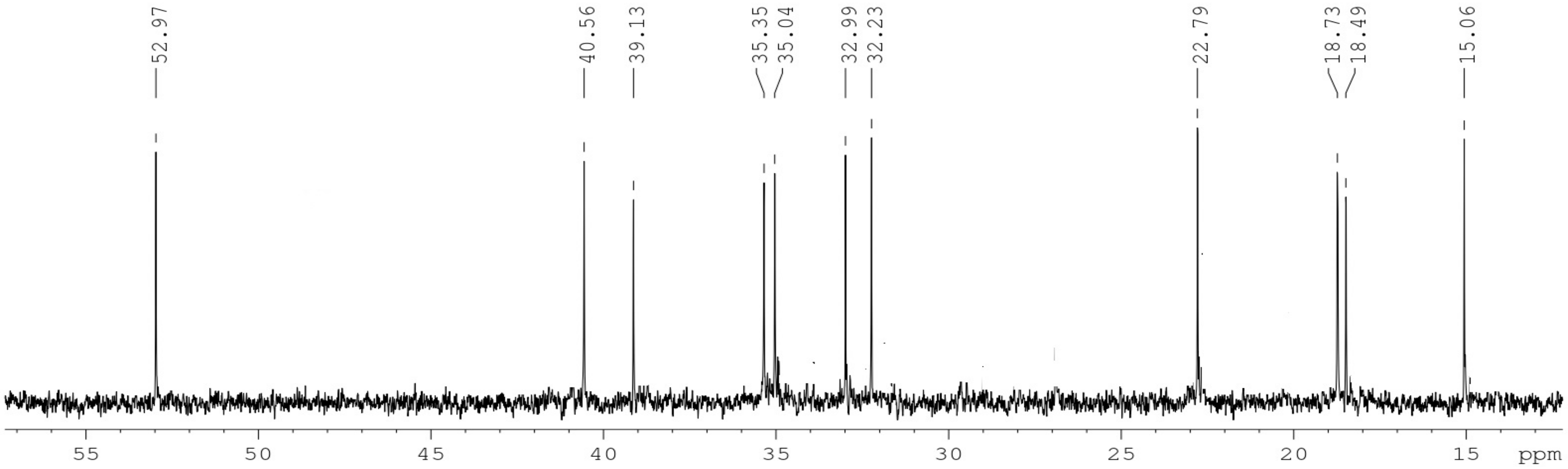

^{1}H NMR spectrum of compound 18

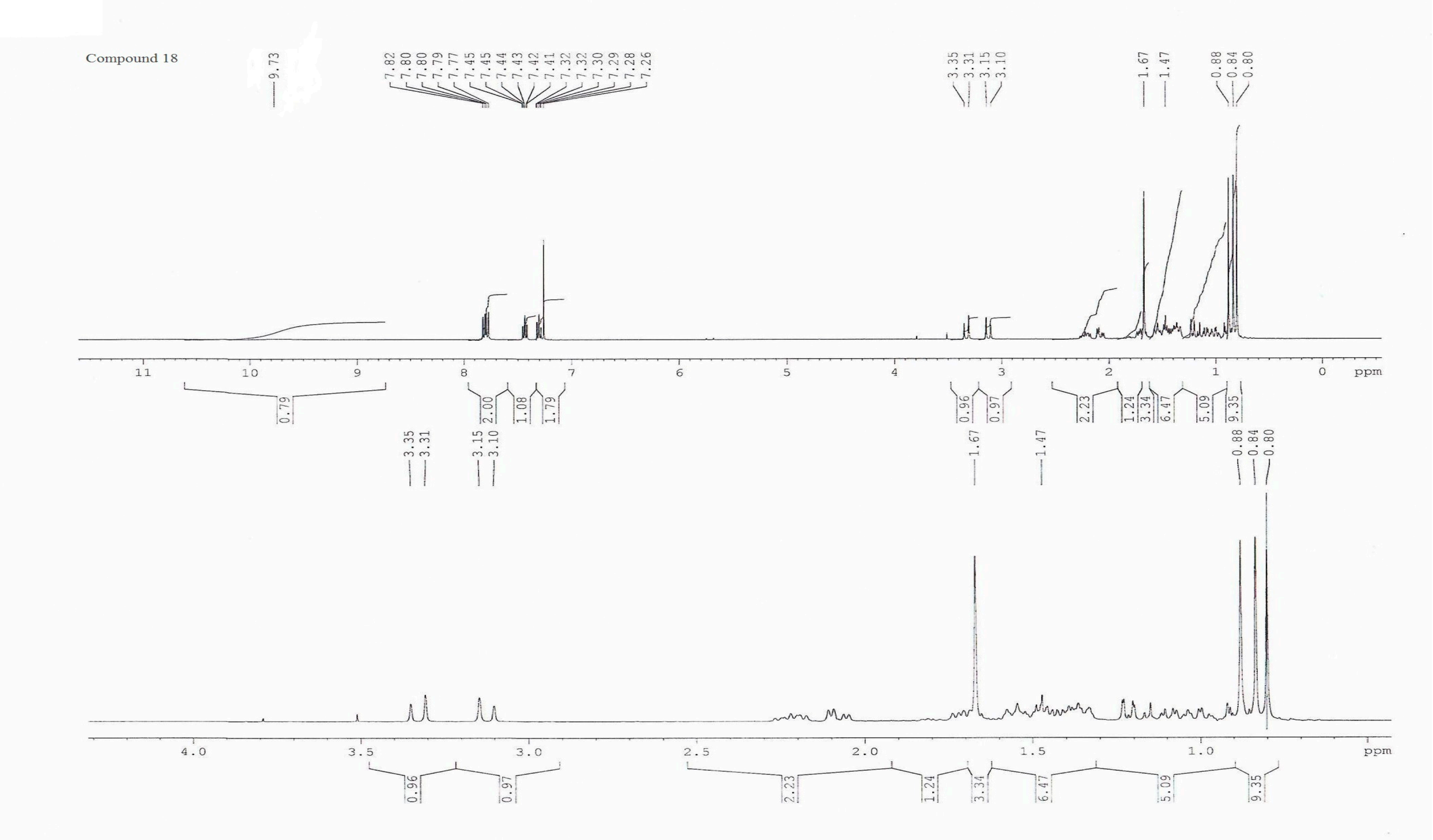

^{13}C NMR spectrum of compound 18

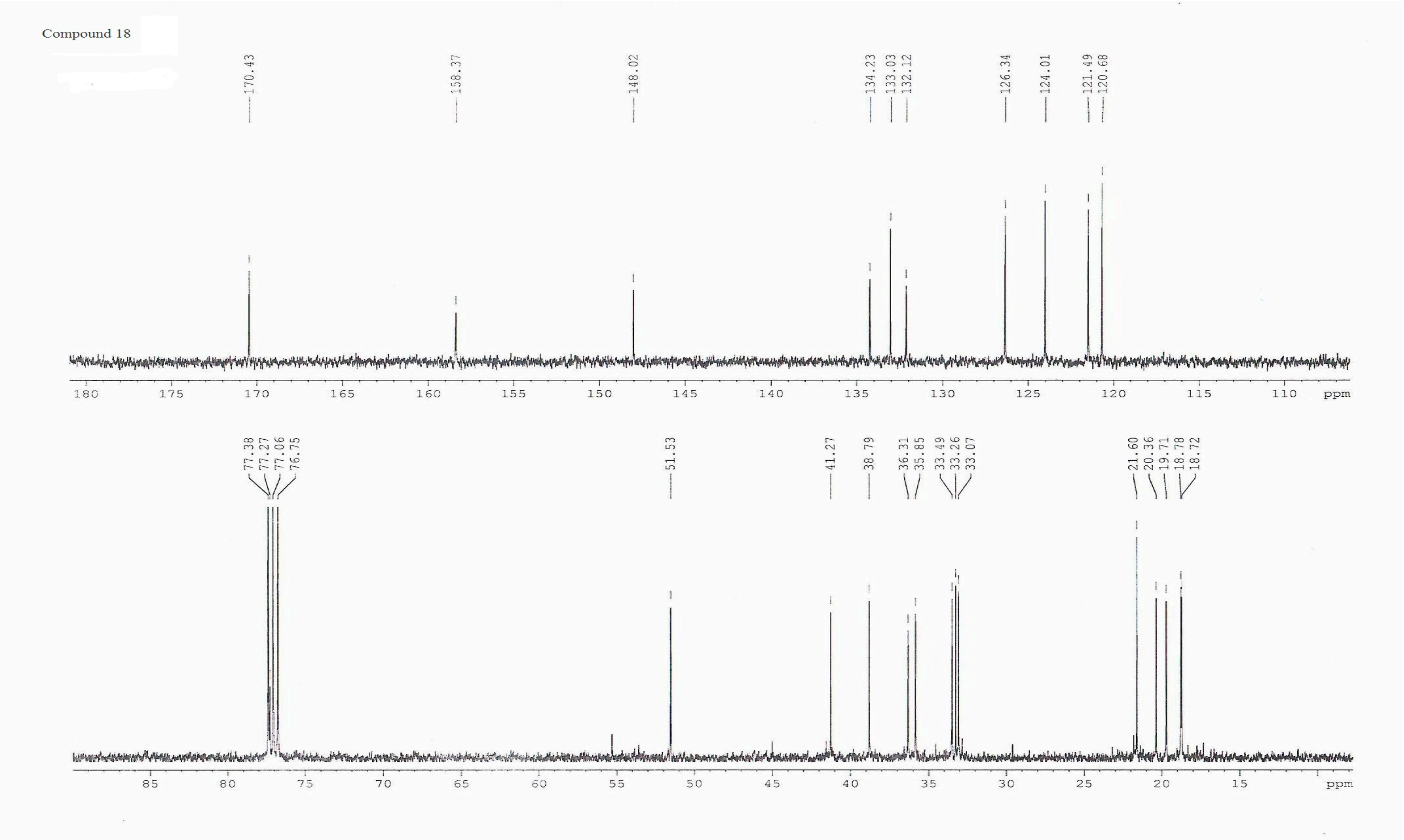

^{1}H NMR spectrum of compound 21

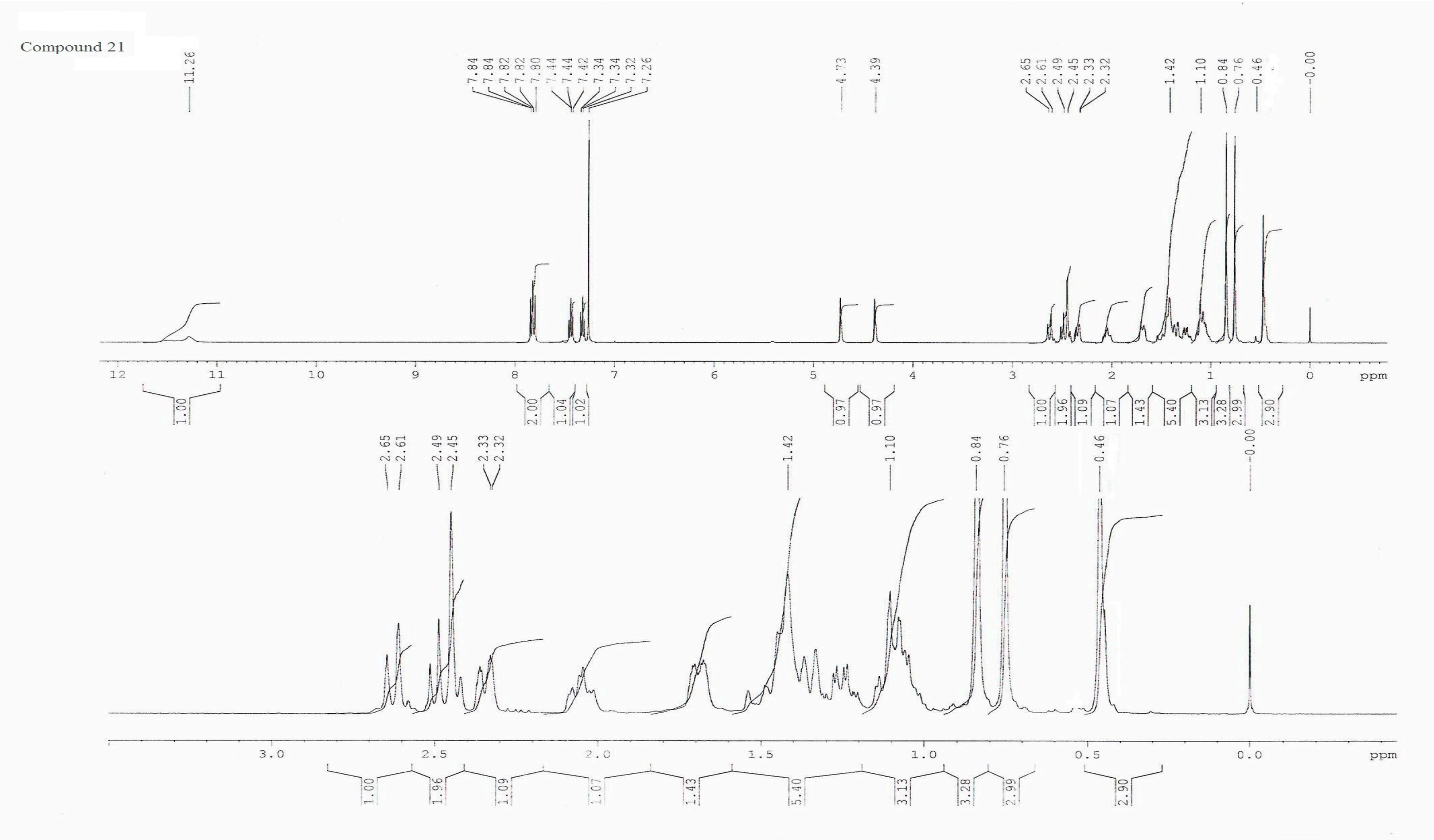

^{13}C NMR spectrum of compound 21

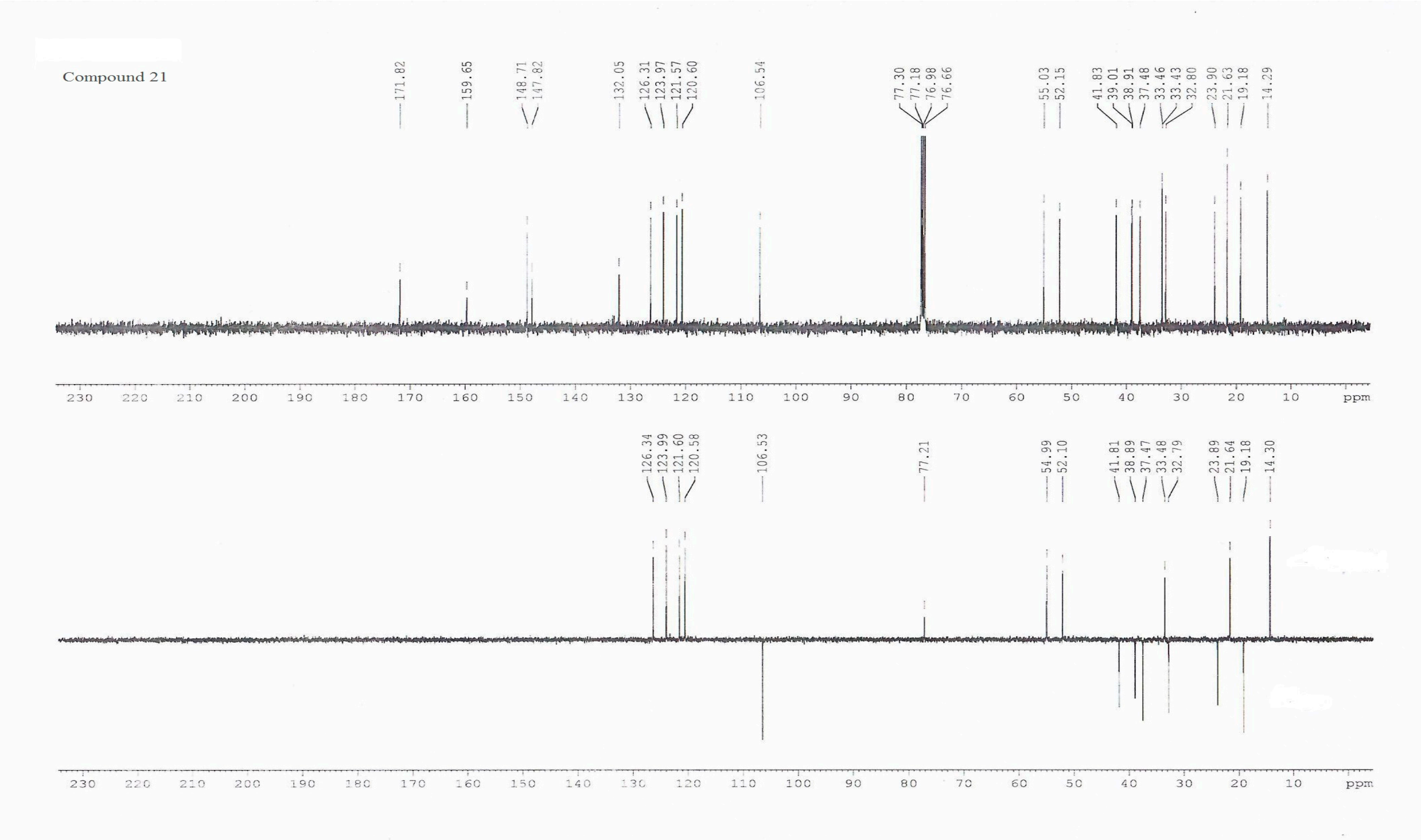

¹H NMR spectrum of compound 22

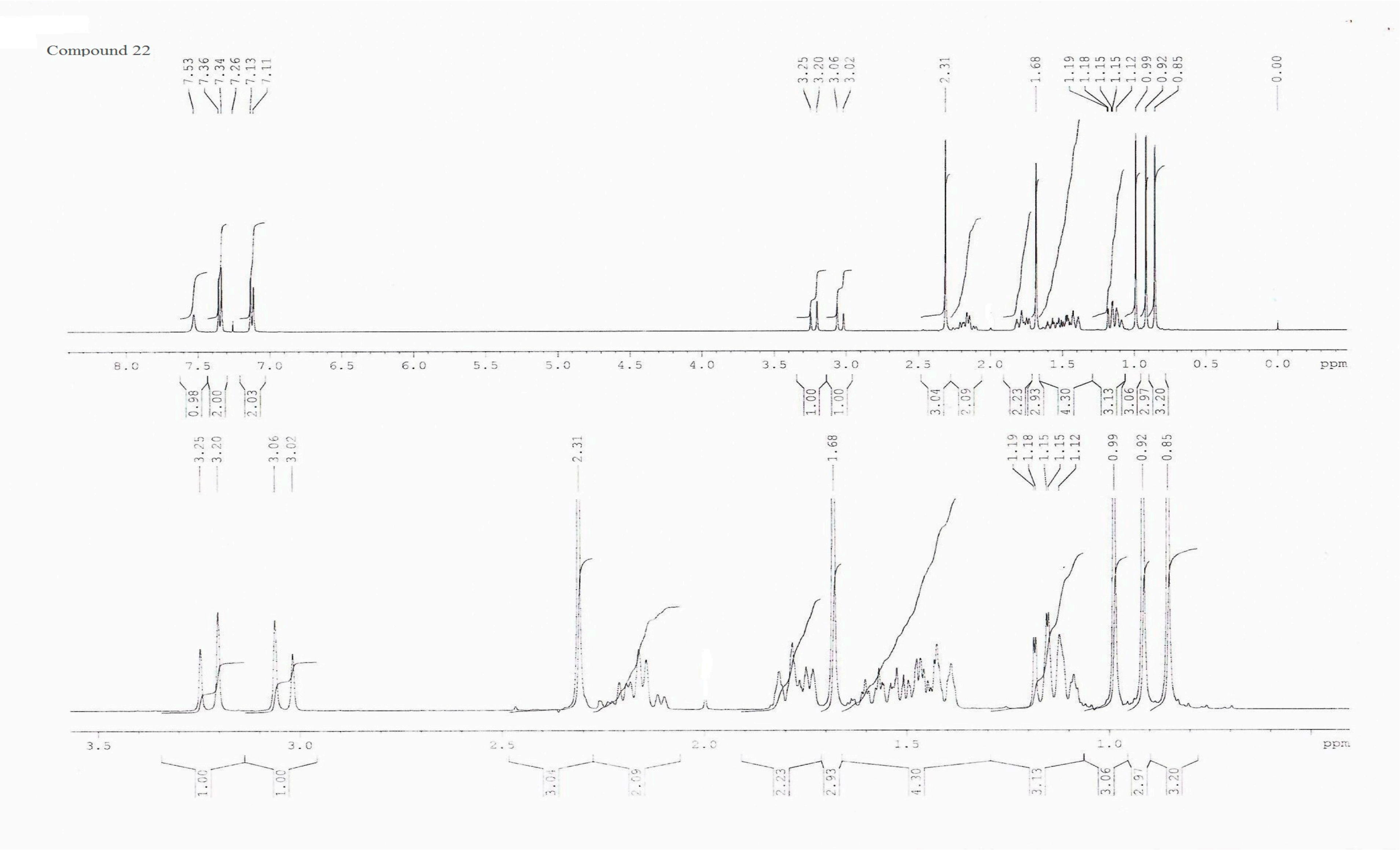

^{13}C NMR spectrum of compound 22

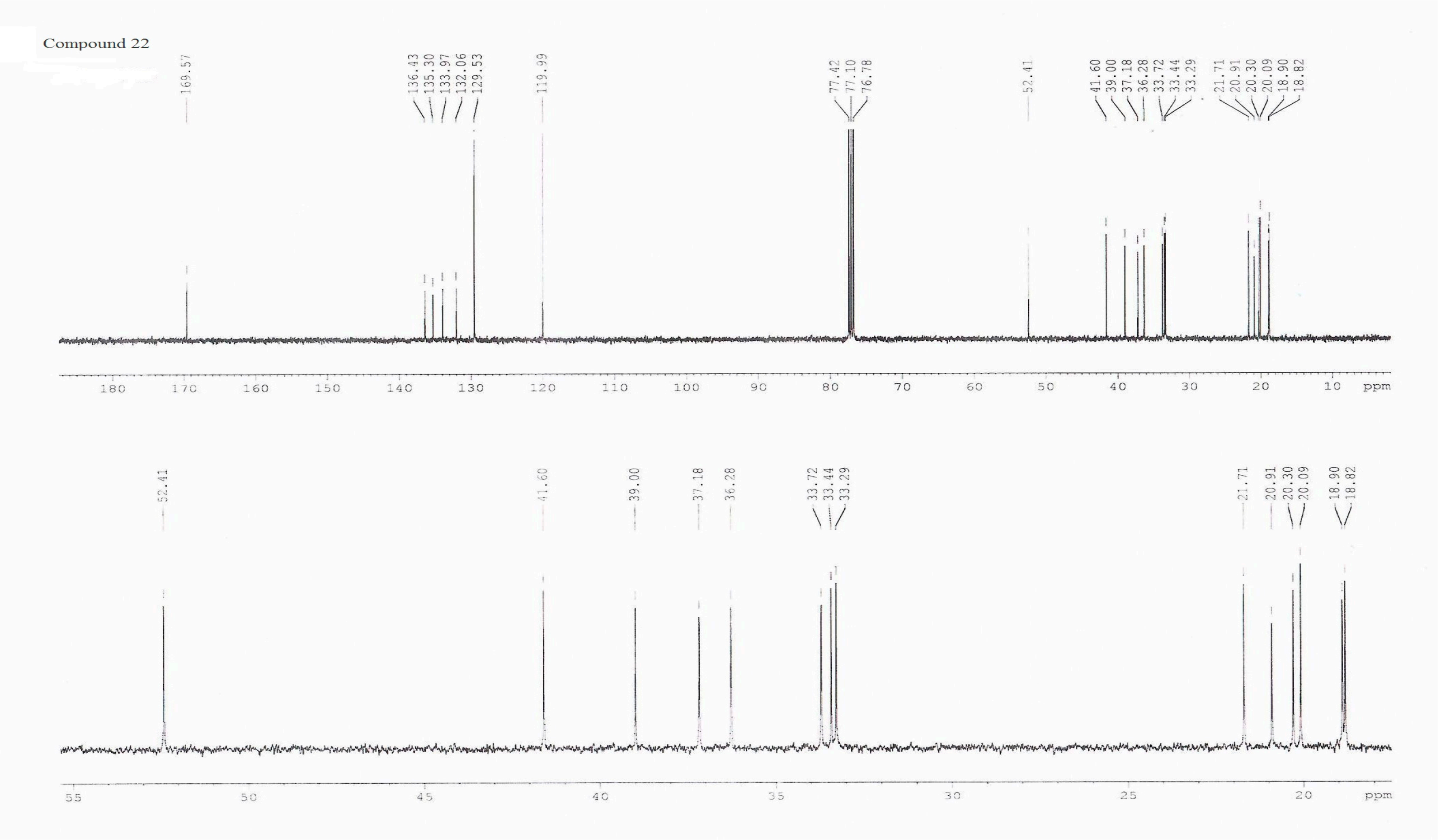

^{1}H NMR spectrum of compound 23

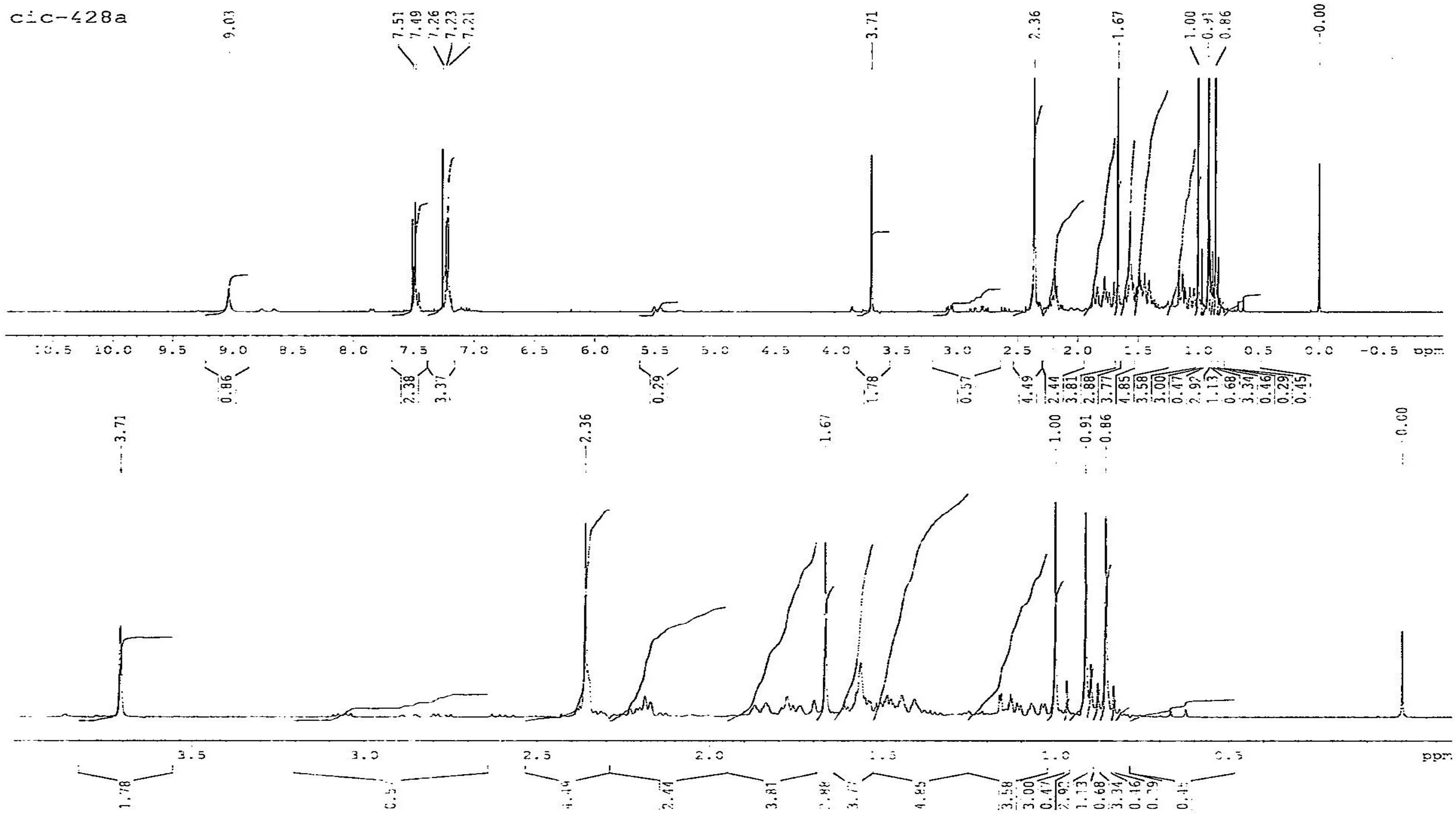

^{13}C NMR spectrum of compound 23

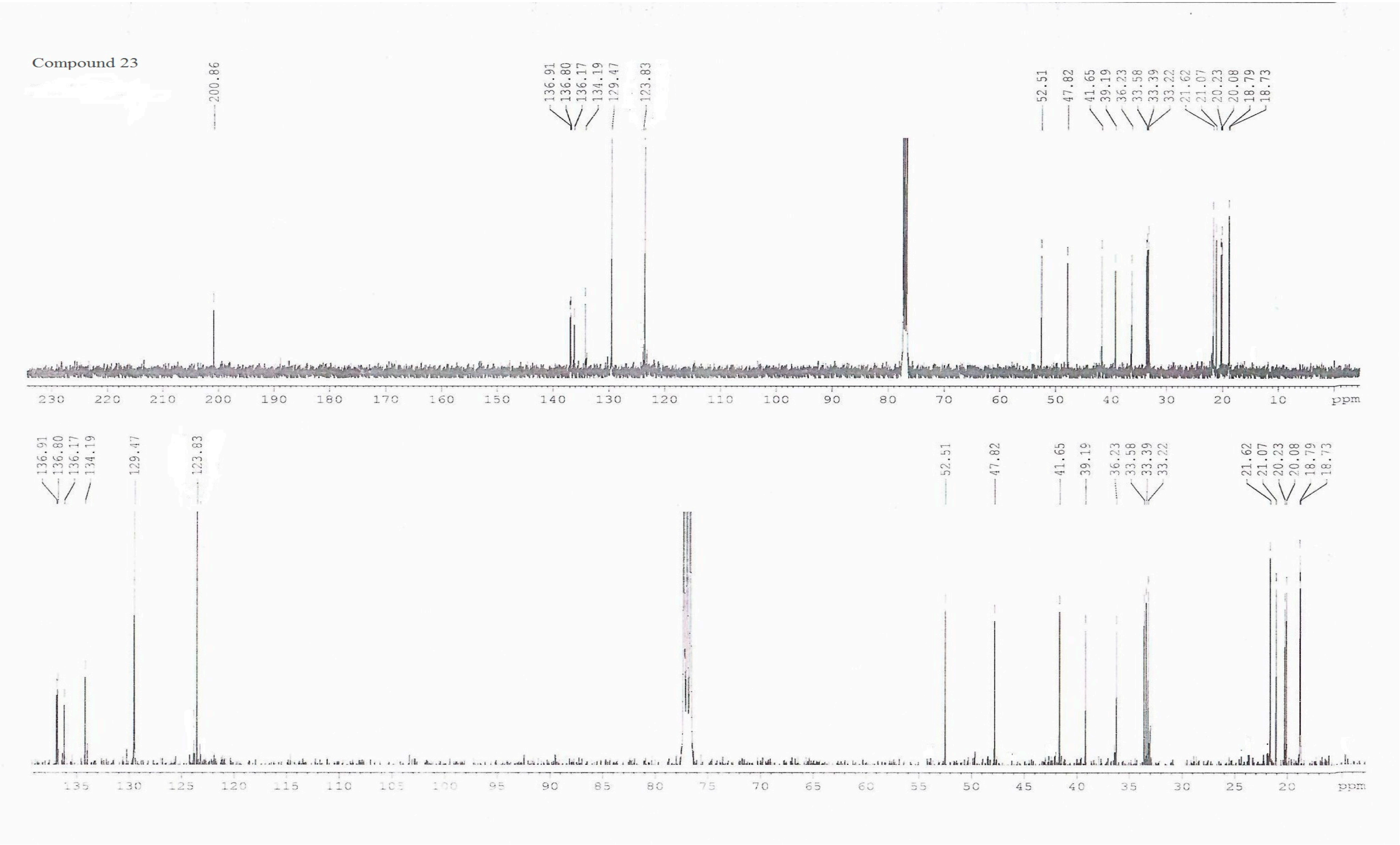

^{1}H NMR spectrum of compound 24

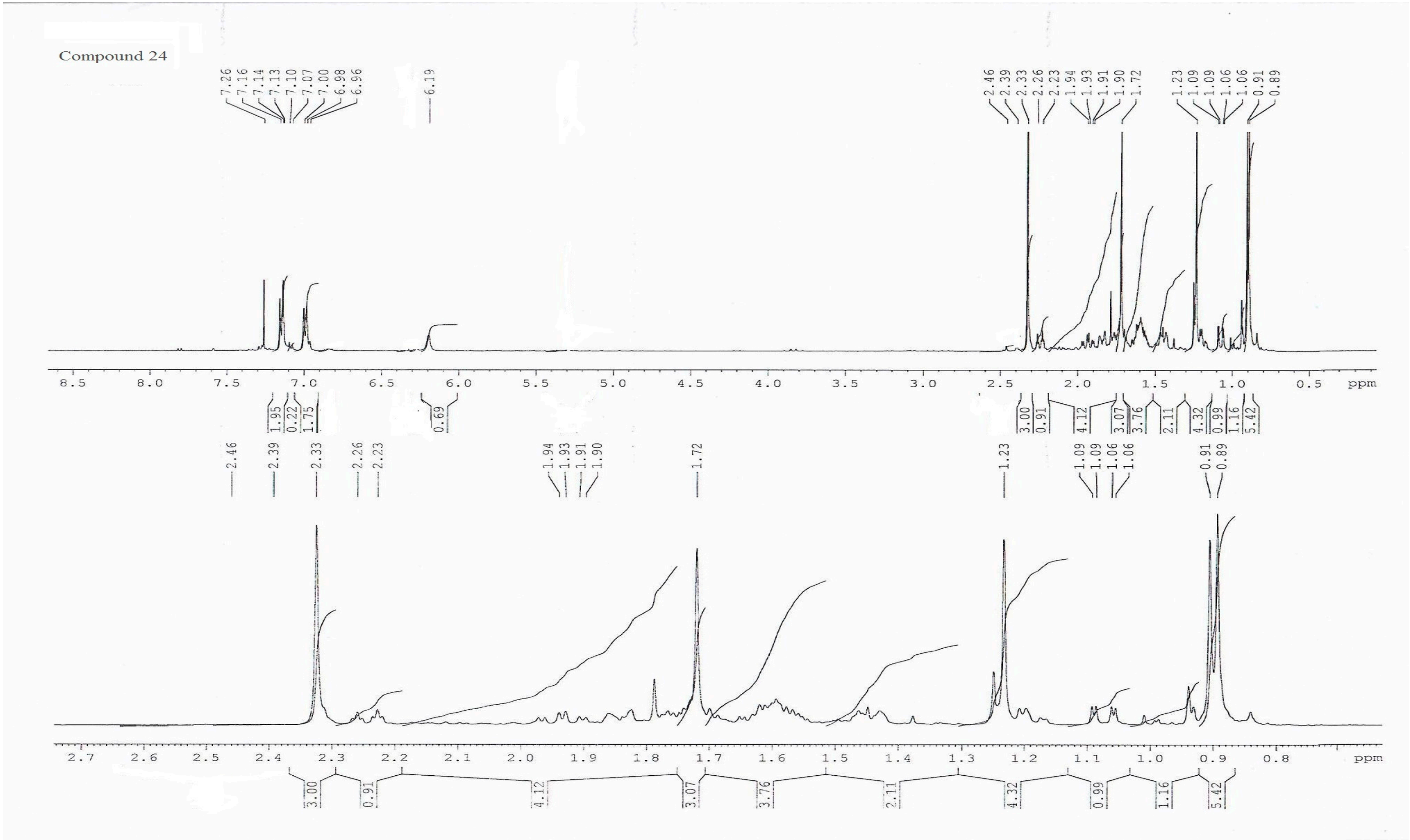

^{13}C NMR spectrum of compound 24

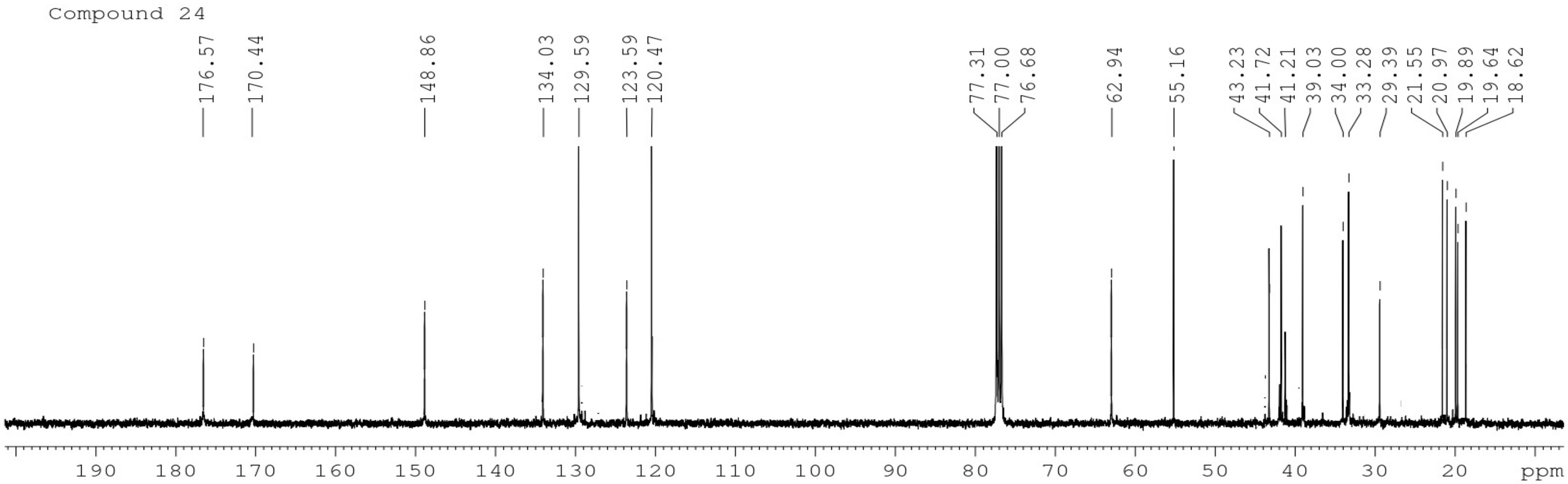

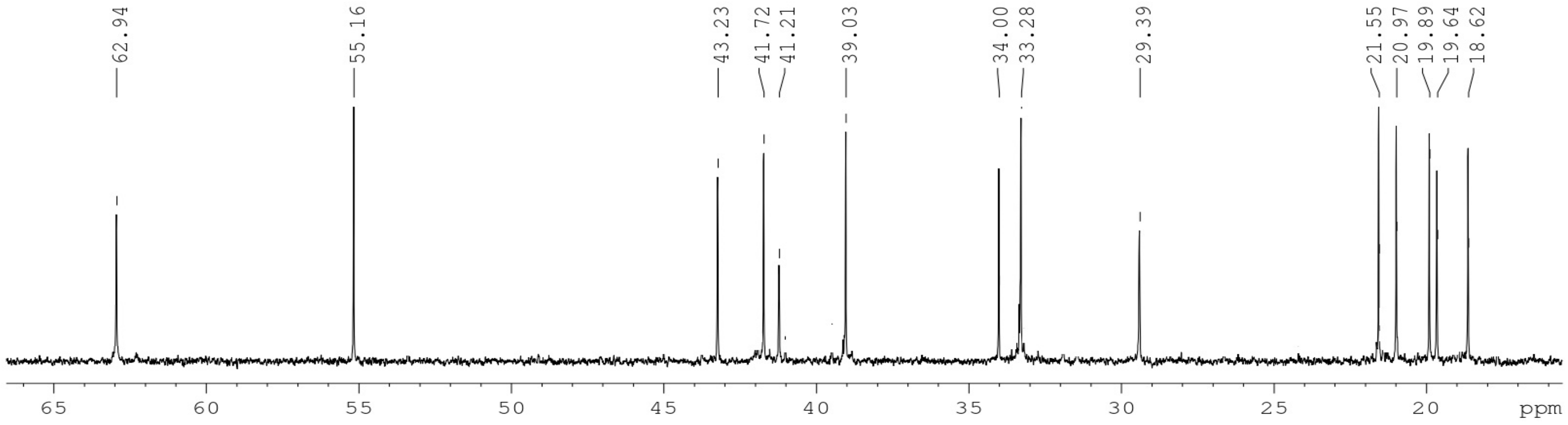

^{1}H NMR spectrum of compound 25

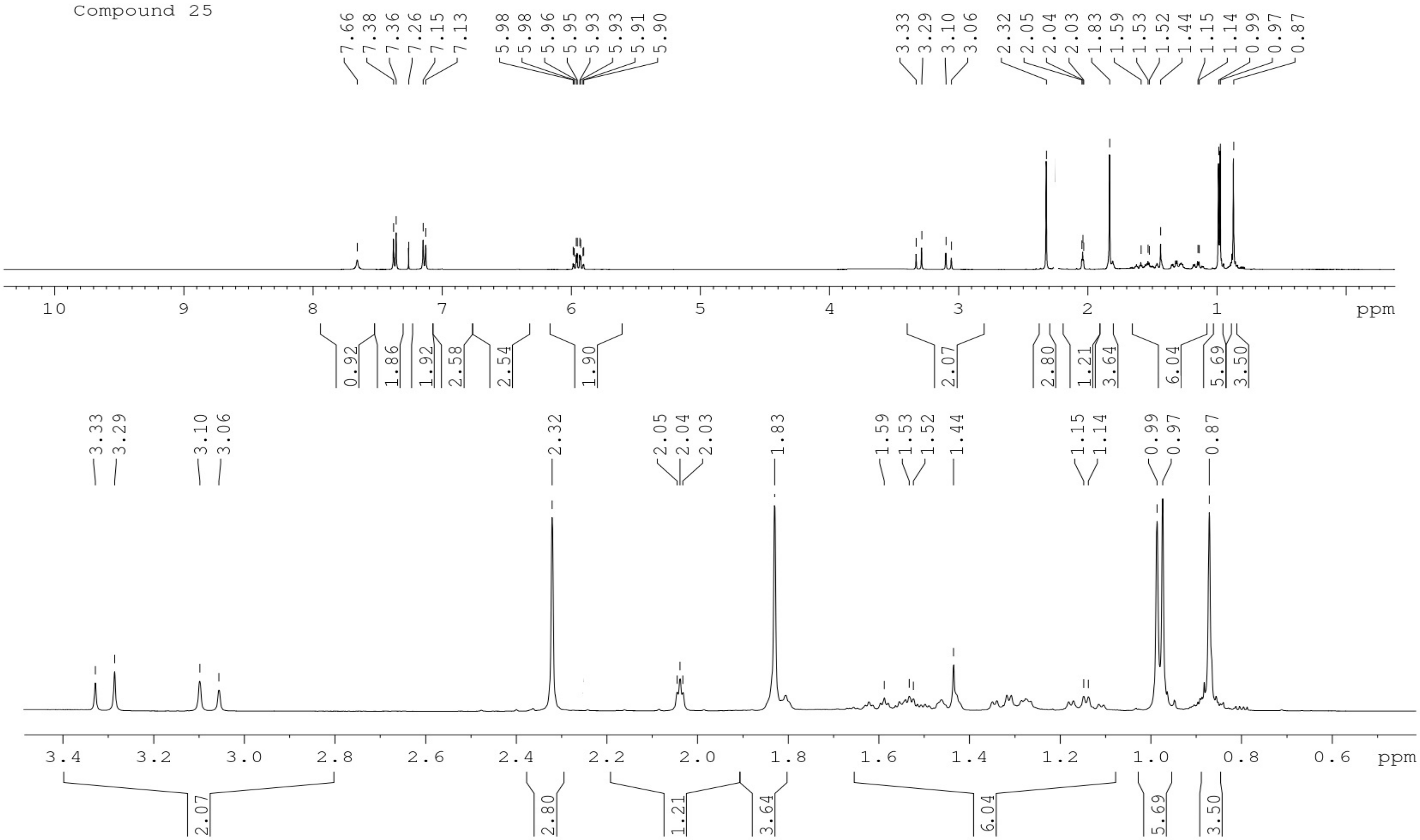

^{13}C NMR spectrum of compound 25

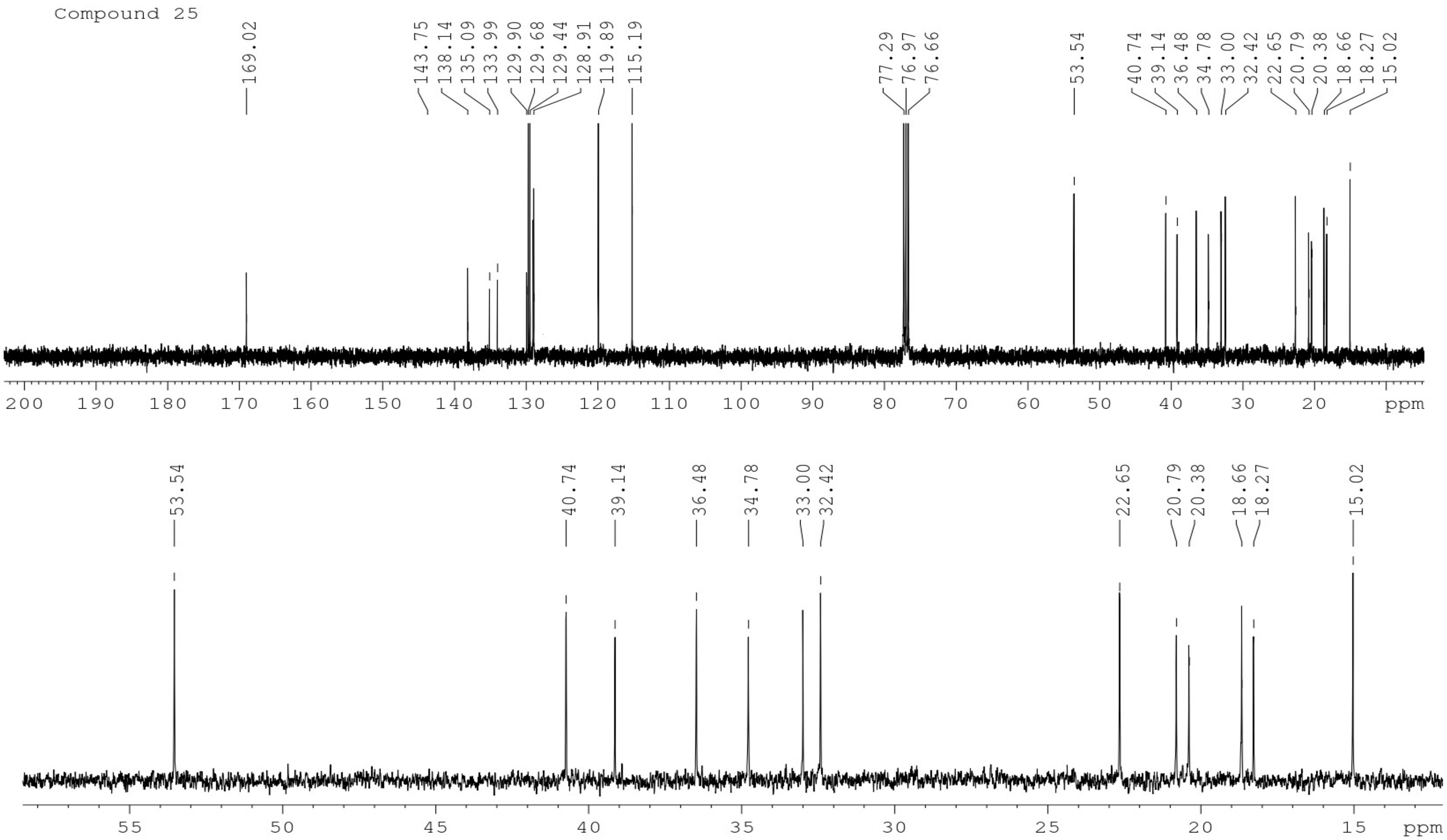

^{1}H NMR spectrum of compound 26

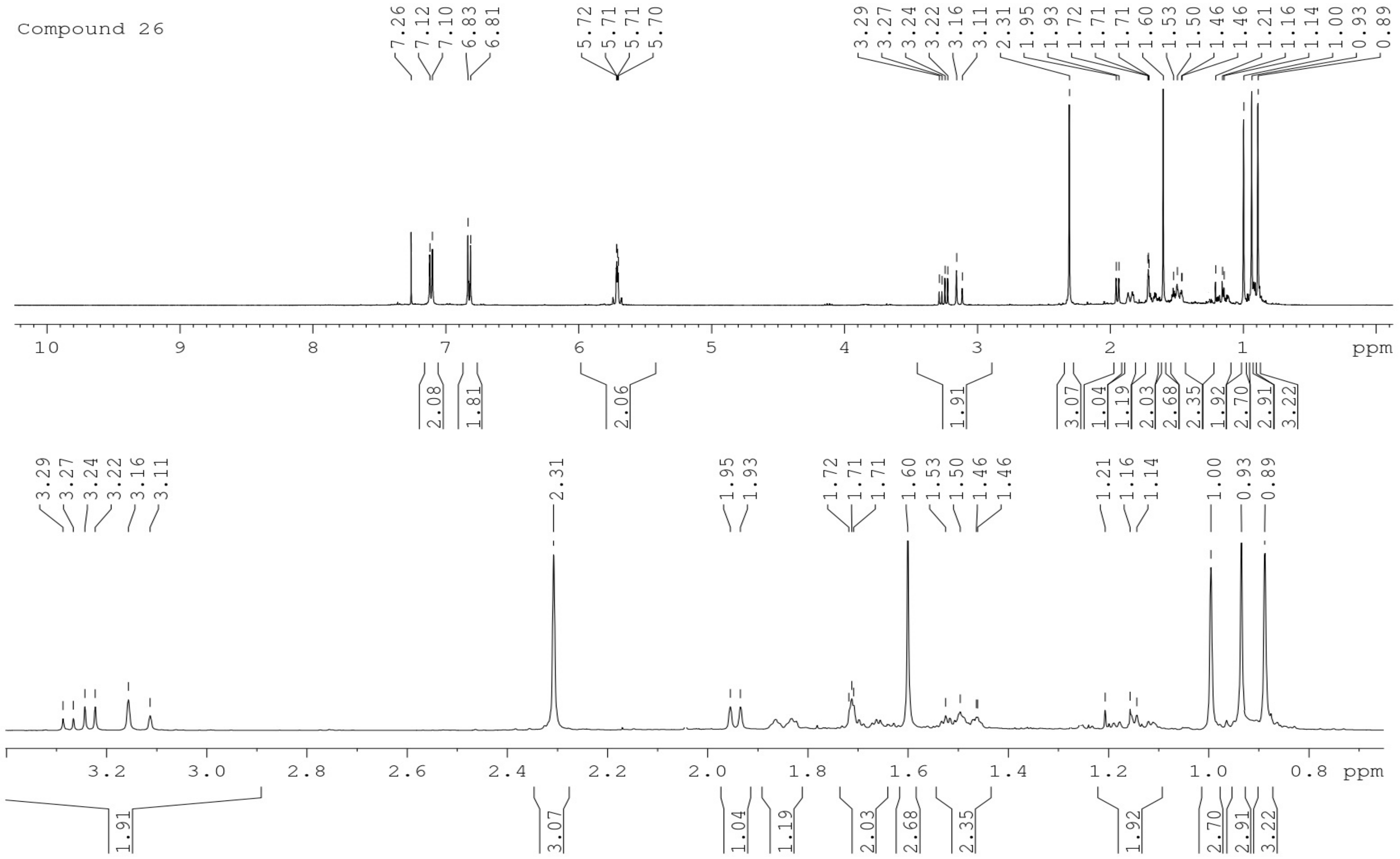

^{13}C NMR spectrum of compound 26

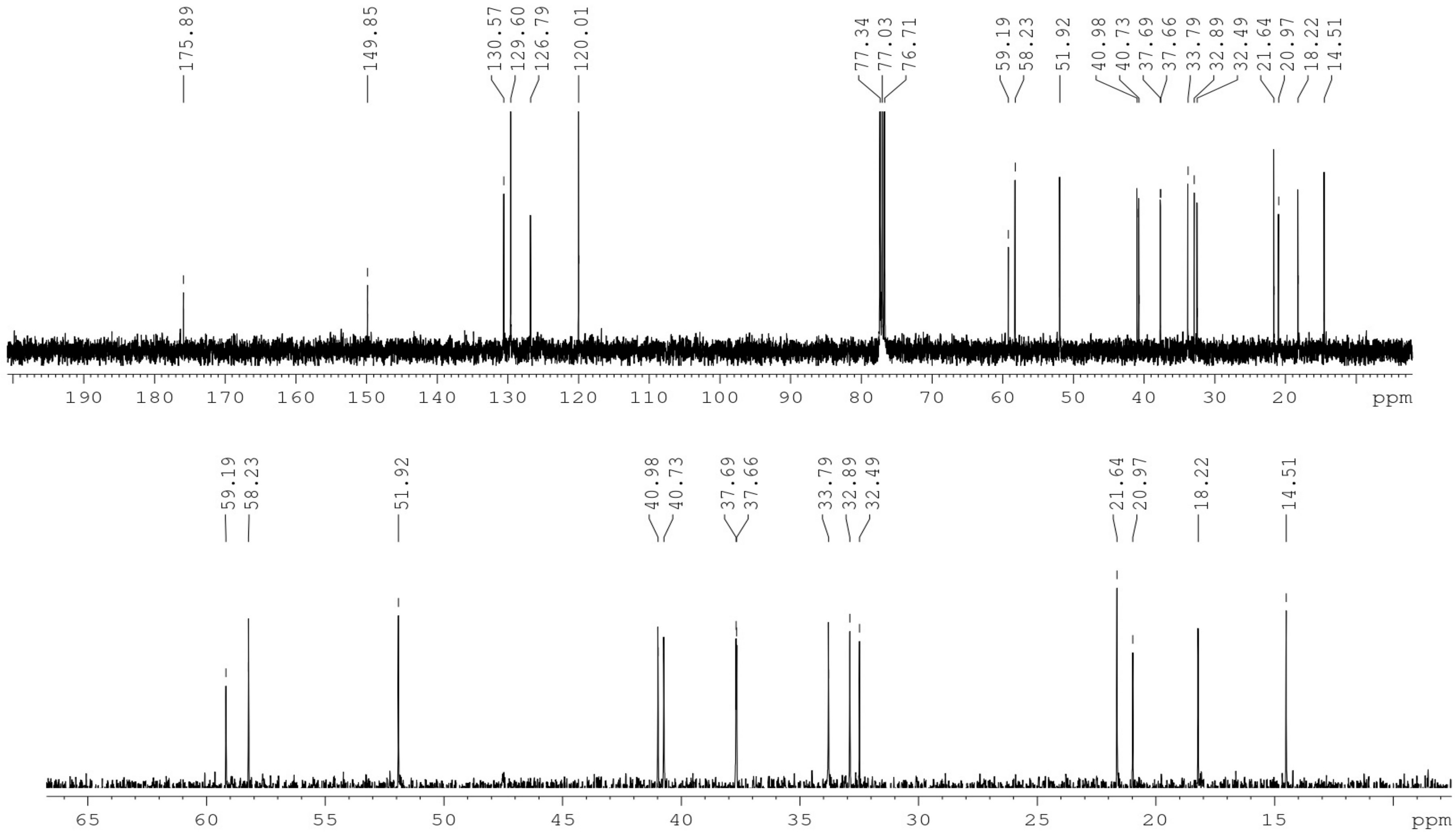

^{1}H NMR spectrum of compound 27

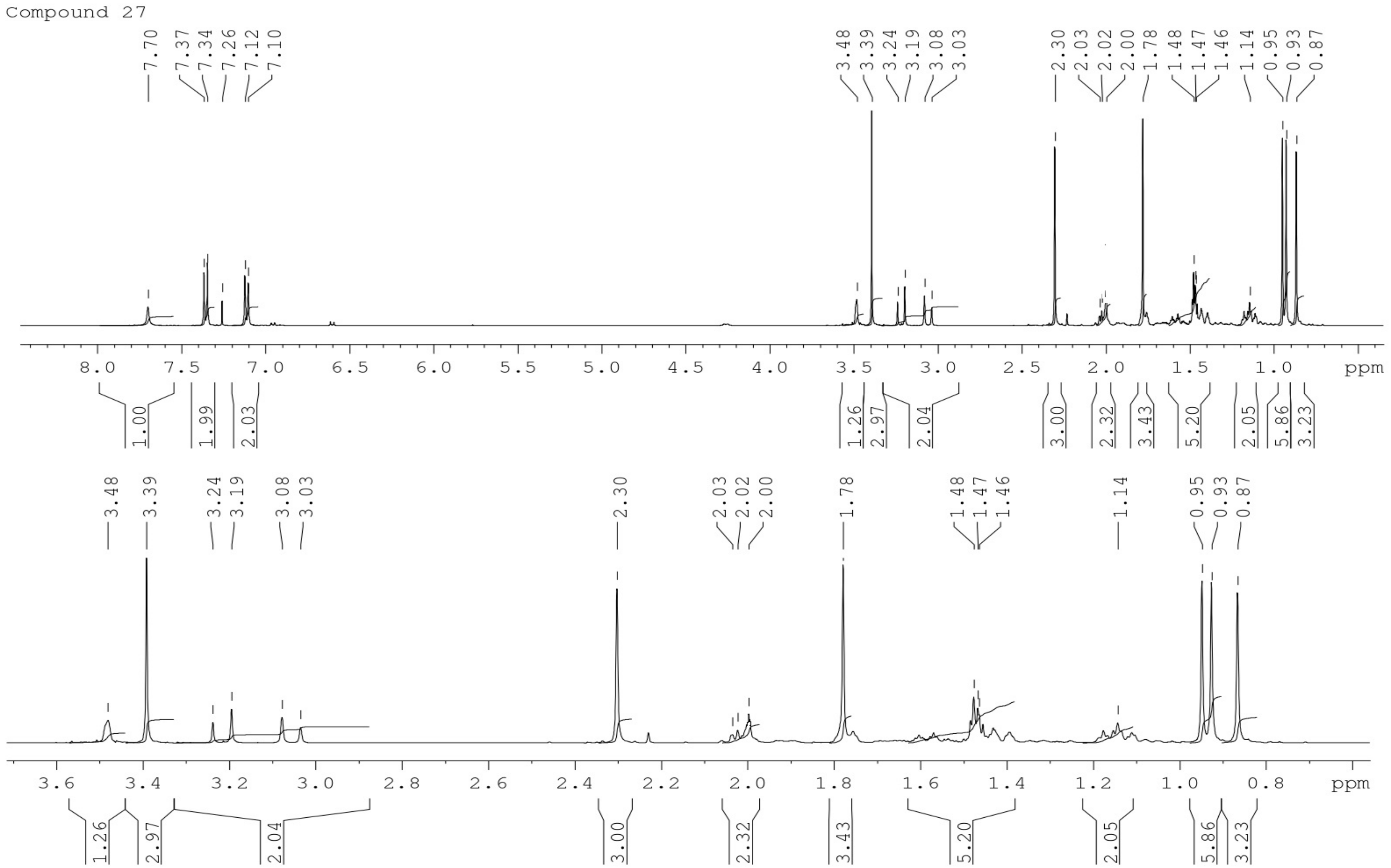

^{13}C NMR spectrum of compound 27

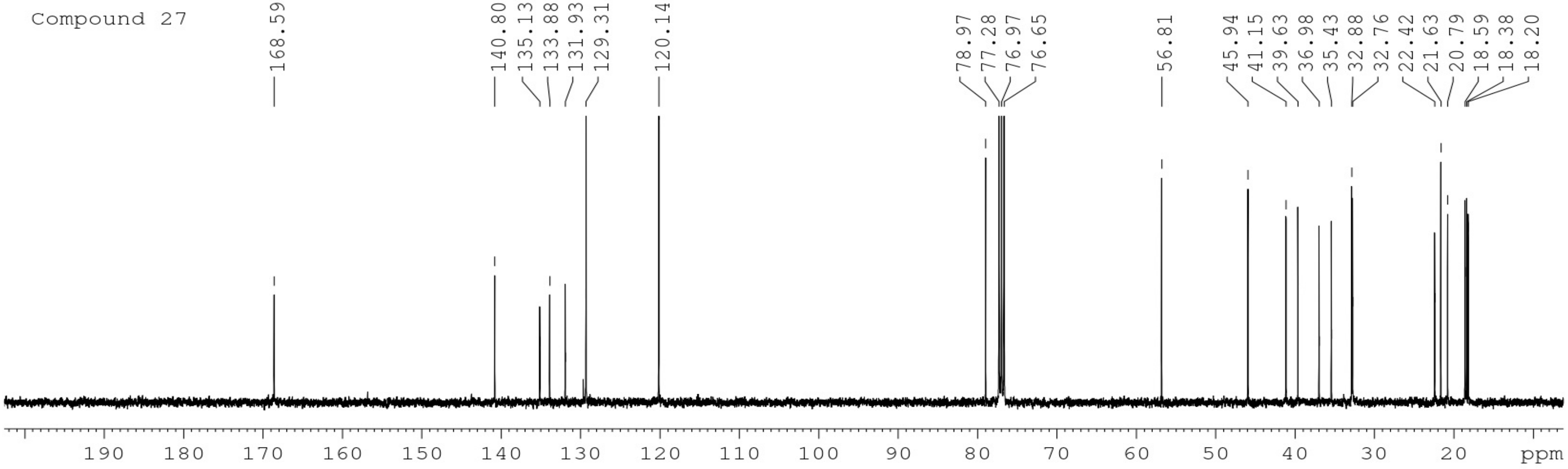

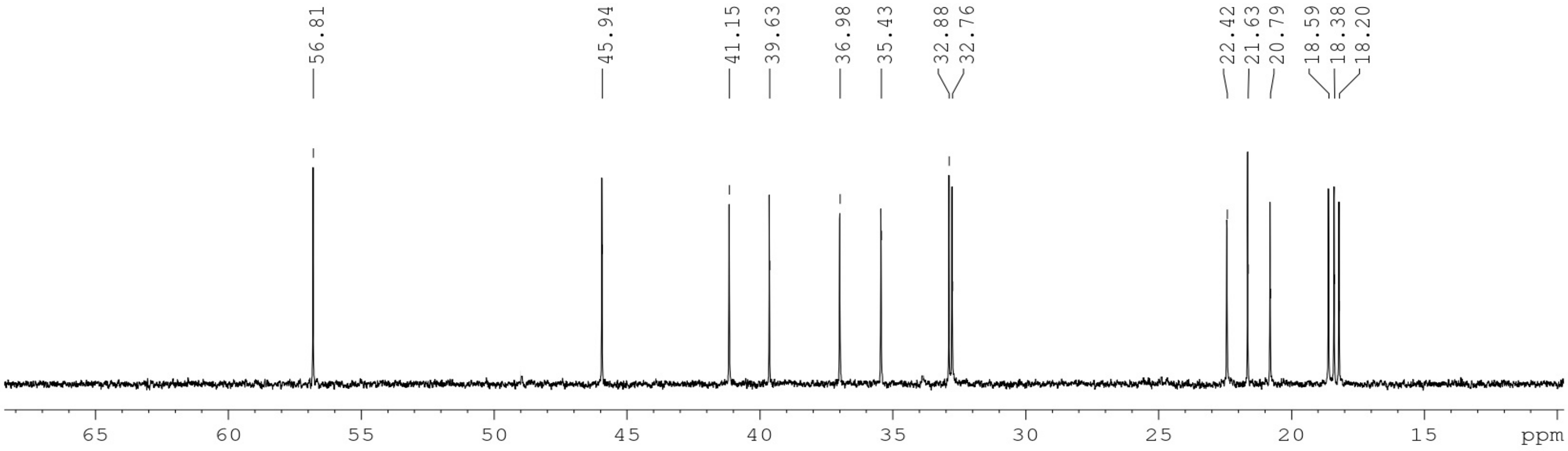

Supplement: Supplementary file 1 [file molecules-27-05082-s001.zip › molecules-1813495-supplementary.pdf]
